# Analysis of the role of retrotransposition in gene evolution in vertebrates

**DOI:** 10.1186/1471-2105-8-308

**Published:** 2007-08-24

**Authors:** Zhan Yu, David Morais, Mahine Ivanga, Paul M Harrison

**Affiliations:** 1 Department of Biology, McGill University, Stewart Biology Building, 1205 Docteur Penfield Ave., Montreal, QC, H3A 1B1 Canada

## Abstract

**Background:**

The dynamics of gene evolution are influenced by several genomic processes. One such process is retrotransposition, where an mRNA transcript is reverse-transcribed and reintegrated into the genomic DNA.

**Results:**

We have surveyed eight vertebrate genomes (human, chimp, dog, cow, rat, mouse, chicken and the puffer-fish *T. nigriviridis*), for putatively retrotransposed copies of genes. To gain a complete picture of the role of retrotransposition, a robust strategy to identify putative retrogenes (*PRs*) was derived, in tandem with an adaptation of previous procedures to annotate processed pseudogenes, also called retropseudogenes (*RψGs*). Mammalian genomes are estimated to contain 400–800 *PRs *(corresponding to ~3% of genes), with fewer *PRs *and *RψGs *in the non-mammalian vertebrates. Focussing on human and mouse, we aged the *PRs*, analysed for evidence of transcription and selection pressures, and assigned functional categories. The *PRs *have significantly less transcription evidence mappable to them, are significantly less likely to arise from alternatively-spliced genes, and are statistically overrepresented for ribosomal-protein genes, when compared to the proteome in general. We find evidence for spurts of gene retrotransposition in human and mouse, since the lineage of either species split from the dog lineage, with >200 *PRs *formed in mouse since its divergence from rat. To examine for selection, we calculated: *(i) *K_a_/K_s _values (ratios of non-synonymous and synonymous substitutions in codons), and *(ii) *the significance of conservation of reading frames in *PRs*. We found >50 *PRs *in both human and mouse formed since divergence from dog, that are under pressure to maintain the integrity of their coding sequences. For different subsets of PRs formed at different stages of mammalian evolution, we find some evidence for non-neutral evolution, despite significantly less expression evidence for these sequences.

**Conclusion:**

These results indicate that retrotranspositions are a significant source of novel coding sequences in mammalian gene evolution.

## Background

Genes are subject to many different processes that give rise to novel sequences, such as segmental and local duplication, gene conversion, and retrotransposition. The extent to which these different processes contribute to gene evolution is unclear. In the present paper, we focus on the phenomenon of *gene retrotransposition*. Retrotransposition entails the reverse transcription of an mRNA transcript and the subsequent re-integration of the resulting cDNA into genomic DNA, in germ-line cells [1]. There is substantial genomic evidence for large-scale retrotransposition of mRNAs in mammalian genomes, from detection of thousands of apparent retropseudogenes in human, mouse and rat [2-4]. Such retropseudogenes (*RψGs*) are decayed or disabled gene sequence copies (typically bearing frameshifts and stop codons) that demonstrate the hallmark characteristics of retrotransposition, namely lack of introns of the parental gene, and also 3' polyadenine tails, if formed more recently [5]. Other features include short direct repeats flanking the sequence (for young retrotranspositions) [6], frequent 5' truncations, and genomic location different from that of the parent gene [2,3]. It has been demonstrated experimentally that *RψG*s can be formed through the action of LINE-1 reverse transcriptases [7]. The computational comparison of LINEs and *RψG*s also supports the generation of *RψG*s by LINEs [8]. The poly(A) tails and frequent truncations found at the 5' end in the *RψG*s are typical for LINEs [2]. Moreover, they share similar structures, including a common TT|AAAA insertion motif [8].

Since the substantial majority of these retrosequences bear disablements (frameshifts and stop codons), or have codon substitution patterns indicative of decay [9,5,3], gene retrotransposition appears generally to lead to non-functional sequences that decay in the genomic DNA as evolution progresses [10,2,9]. However, even though the promoters of these gene retrosequences are not transferred, a small minority of them appears to be transcribed [11]. For the human genome, there is a small population of at least ~200 transcribed processed pseudogenes, which have the symptoms of a lack of coding ability despite evidence of transcription, and are significantly likely to be found near others genes (as would be expected if they are co-opting promoters) [11].

Generation of a new functional gene is also a possible outcome of retrotransposition [10]. There is an increasing number of transcribed, functionally characterized genes in mammalian and invertebrate animal genomes reported to bear the characteristics of retrosequences [12]. Over ninety such *retrogenes *have been annotated in the human and mouse genomes [13]. Most of the functional retrogenes identified are expressed in testis and may have provided important raw material for rapid testis evolution in primates [12].

Here, to derive an overview of the role of *gene retrotransposition *in the genome evolution of vertebrates, and particularly mammals, we derive and apply a robust procedure to annotate gene retrotranspositions, built on our previous analyses of retropseudogenes [11,3,2,14]. Our strategy incorporates a new rapid procedure for annotating retrocopies in the genomic DNA, in tandem with a pipeline to identify them in existing gene annotations. This PR annotation pipeline incorporates aging of the sequences through evolutionary rate analysis relative to putative parents and their orthologs, as well as analysis of the chromosomal milieus of these sequences and their putative parents. We find evidence for, on average, several hundred *PRs *in each proteome. Focussing on human and mouse, we find evidence for spurts of gene retrotransposition in both human and mouse, since divergence from dog. A small number (>50) of *PRs *have formed in both mouse and human since divergence form dog, that show signs of being under selection to maintain their coding sequences.

## Methods

### Genome data

**Figure 1:**
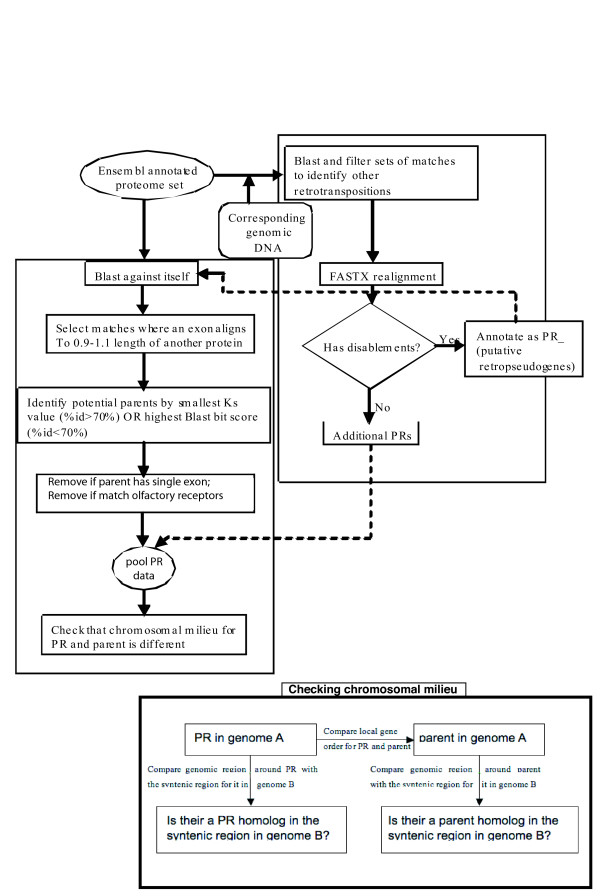
**Pipeline summarizing the annotation of PRs and retropseudogenes**. The pipeline for PR annotation is summarized. There is an inset at the bottom, that summarizes the tests for local gene order and chromosomal milieu.

**Figure 2:**
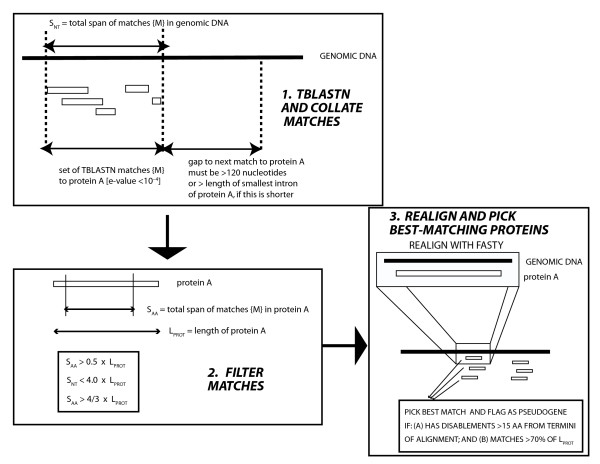
**Rapid annotation of retropseudogenes**. (1) TBLASTN matches (e-value ≤ 10^-4^) of the annotated proteome against the genomic DNA are sorted by coordinates and collated for each protein to form a set of matches {M}. (2) The sets {M} are filtered using length-based heuristics. (3) Each protein is realigned to the genomic DNA using FASTY, and the best-matching proteins at each point have disablements and that matches >70% of the length of the parent sequence are picked as retropseudogene annotations.

The genome sequences and annotations of seven organisms analyzed in this paper (human, dog, cow, mouse, rat, chicken and *Tetraodon nigriviridis*), were downloaded from the Ensembl Web site [15], in January 2005. Version 2.1 of the chimpanzee assembly (downloaded in April 2006) was also used. Putative retrogenes (*PRs*) were identified in the annotated proteomes of eight vertebrates using the pipeline in Figure 1. This procedure is described in detail below. In tandem, putative retropseudogenes (*RψGs*), and additional *PRs *outside of current protein annotations, were assigned using a modification of previous procedures (Figures 1 &2) [14,2]. Genes from which *PR*s and *RψG*s are thought to have originated are called *parent genes*.

### Rapid identification of retropseudogenes (*RψGs*)

Retropseudogenes were annotated on the Ensembl genome versions used in this present analysis, using the rapid improvement of previous procedures to identify retropseudogenes described above (summarised in Figure 2) [11,5,14,2,3].

### Identification of putative retrotransposed genes (*PRs*)

(1) *Homology detection*: Each proteome was compared against itself using BLAST to find similarities with e-value ≤ 10^-4 ^[16]. Any match to a potential pseudogene contaminant in the proteome annotations was removed (Figure 1).

(2) *Exon seam analysis*: Exon boundary information for each protein was extracted from the appropriate Ensembl genome annotation files. The positioning of exon boundaries in encoded protein sequences, *i.e*., 'exon seams', was then deduced [11]. Using the positioning of exon seams, the BLAST matches between proteins were filtered to pick out alignments between a protein encoded by a multiple-exon gene, and a single exon of another gene. To define *PRs*, the length of the exon was required to be between 0.9 and 1.1 of the whole length of the multiple-exon protein. (This is stricter than the criterion of 0.7 of the length used for annotation of retropseudogenes [2]).

*(3) Assignment of parent genes: *To assign parent genes to *PRs*, we calculated substitution rates at synonymous codon sites (*i.e*., Ks values) for all matches to *PRs *using the package PAML, for all instances where the amino-acid sequence identity for the pair of sequences is ≥ 70%. The sequence with the smallest Ks value was chosen as the 'parent gene'. For sequence identities <70%, saturation of substitutions is likely [17], and so the sequence with the highest BLAST bitscore in alignment with the *PR *was chosen as the putative parent.

(4) *Additional filtering: *In addition, *PRs *were discarded if they matched olfactory receptors (ORs) with BLAST e-value ≤ 10^-4 ^over ≥ 0.5 of the length of the OR, since recent olfactory receptors (ORs) have probably originated from different mechanism other than retrotransposition [2]. Olfactory receptor sequences were taken from ORDB [18].

(5) *Local gene order test for similarity of the chromosomal milieus for PRs and parent genes: *To check that the *PRs *did not arise from local or segmental duplication, we derived a 'local gene order' test. For this test, we compared the chromosomal milieus of *PRs *and parents for significant similarity, as follows. Proteins encoded by genes adjacent to *PRs *in the chromosomes were BLASTed against the corresponding proteins from genes adjacent to putative parents, for a given window (*w*_*genes*_) of number of genes in either direction (5' and 3'). A *w*_*genes *_size of 7 (the *PR *or parent, plus 3 genes in either direction), with an allowance for one gap between the positions of matches within *w*_*genes*_, was found to be suitable. The number of significant homologous matches *N*_*homologs *_(BLAST e-value ≤ 10^-4^, sequence identity >40%, and match ≥ 0.6 length of both *PR *and parent) between the milieus of *PR *and parent was tallied. An expected benchmark distribution for *N*_*homologs *_was derived for the chromosomal milieus of 1,000 randomly-sampled pairs of proteins that have any significant BLAST match to each other (e-value ≤ 10^-4^). From examination of this distribution, we found that 80% of such random pairs have *N*_*homologs *_<1, and 87% have *N*_*homologs *_≤ 1. We thus chose *N*_*homologs *_= 1, as a suitable threshold for local similarity arising from duplication of genomic DNA. However, the results differ little if a threshold of *N*_*homologs *_= 0 is used. This procedure was applied to the genomes of the human and mouse. Interestingly, application of this criterion resulted in the exclusion of many sequences with large individual exons (*PRs *with *FLE*_*parent *_≥ 0.8; 36/86 = 42% of those excluded), that may be false positives in our data set of *PRs*. A large fraction of these sequences (68%) tend to have long, tandem arrays of Zn-finger domains covering more than a third of their sequences (Additional Figure 1).

### Additional filtering and annotation

The following additional analysis was performed on the *PRs*:

*(i) Fraction of largest exon in parent: *We calculated the fraction of the length any parent gene that is taken up by its largest exon. This is denoted *FLE*_*parent*_. We found that there is no peculiar tendency for the parents of *PRs *to have a single large exon (which would yield a tendency for high FLE values) [Additional Figure 1].

*(ii) Overlap with retropseudogenes annotations: *Retropseudogenes were annotated on the Ensembl genome versions used in this present analysis, using the rapid improvement of previous procedures to identify retro(pseudo)genes described above. Any PR that overlapped one of these annotations was flagged (Table 1).

**Table 1:** Overview of gene retrotransposition analysis for eight vertebrates

**Species**	Number of genes *	**Number of PRs**	PR matches retrotransposed TE **	**PR overlaps pseudogene annotation**	FLE ≤ 0.8 ***	**Number of retro- pseudogenes (*RψGs*)**	**PRs passing local gene order test**	Matching Refseq mRNA or Unigene consensus ****
**Human**	22219	631 (3%)	78 (12%)	36 (6%)	504	2493	545	145/631 (23%)
**Chimp**	20980	476 (2%)	17 (4%)	5 (1%)	339	1889	----	
**Dog**	18199	409 (2%)	18 (4%)	25 (6%)	363	3505	----	
**Cow**	23147	790 (3%)	46 (6%)	104 (13%)	479	1996	----	
**Mouse**	25021	663 (3%)	31 (5%)	75 (11%)	518	2969	533	58/663 (9%)
**Rat**	22157	567 (3%)	21 (4%)	62 (11%)	492	4520	----	
**Chicken**	17707	321 (2%)	15 (5%)	26 (8%)	267	720	----	
***Tetraodon ***	28005	227 (1%)	4 (2%)	10 (4%)	203	644	----	

* The number of gene annotations (both those labelled 'known' and 'novel') for the genome version downloaded from Ensembl [[15]; see *Methods *for details].

** TE = transposable element ; see *Methods *for details.

*** FLE = fraction of largest exon ; see *Methods *for details.

**** Number of PRs with complete Refseq mRNAs or complete Unigene consensus sequences (percentage of these in brackets); see *Methods *for details. PRs have significantly much less mapping of this transcription information than the whole annotated proteome for these organisms.

*(iii) Filtering for potential transposable elements (TEs): *Each proteome was compared using TBLASTN to libraries of transposable elements taken from the RepeatMasker distribution [19], using an e-value threshold of ≤ 10^-4^. Any proteins containing SINEs, or near-complete matches to LINEs (≥ 0.8 of their lengths), were labeled as potentially TE-containing.

*(iv) Whether single-exon gene or multiple-exon gene: *The *PRs *were labeled as either *single-exon genes *or part of *multiple-exon genes*.

### Orthologs

Orthologs of parent genes were identified using the bi-directional best hits method, using BLAST (e-value ≤ 10^-4^, amino-acid sequence identity ≥ 40% and requiring the alignment to cover ≥ 0.6 of the lengths of each sequence. The bi-directional best hits method is a common procedure for guarding against considering paralogs.

### Analysis of K_s _and K_a_/K_s _values, and derivation of genome- and lineage-specific gene lists

The package PAML [20] was used to calculate maximum-likelihood Ka, Ks and Ka/Ks values for pairs of sequences (either *PR *versus computed ancestral sequences, *or PR *versus parent). In addition, branch-specific maximum-likelihood Ka/Ks values were calculated for three-way alignments of *PR*, parent and parent's orthologs from another close species.

We applied three different strategies based on analysis of Ks, to determine lists of genome-specific and lineage-specific *PRs*. For example, for the human genome, we calculated *human-specific *lists relative to the chimpanzee genome. Also, we calculated *primate-specific *lists for human *plus *chimpanzee, relative to a mammalian outgroup, such as dog or cow. To determine genome-specific lists of *PRs*, we investigated each of the following three methods ("*parent's ortholog*" refers to the ortholog of the parent in the most closely related genome):

*(1) *The distribution of Ks values for orthologous genes in the two organisms was calculated, and the median value *m *derived from this. If Ks [*PR*←→*parent*] <*m *and Ks [*parent *←→*parent's ortholog*] >*m*, then a *PR *is labeled genome-specific;

*(2) *Secondly, a *PR *could be labeled genome-specific if Ks [*PR *←→*parent*] < Ks [*parent *←→*parent's ortholog*];

*(3) *Thirdly, a *PR *could be labeled genome-specific if, in a three-way tree of *PR*, parent and parent's ortholog, the branch-specific Ks [*PR*] is < (Ks [*parent*] + Ks [*parent's ortholog*])/2 ;

Lineage-specific lists were derived in a similar fashion. Additional Figure 2 shows how these three methods overlap which each other. Based on the overlaps observed, we used Method (3) for further analysis.

### Analysis of reading frame conservation

We assessed the reading frame conservation (RFC) in sequences using simulations of insertion and deletion governed by power-law insertion/deletion (indel) statistics [4]. Power-law statistics for indels were extracted from recently-formed RΨGs having ≥ 85% amino-acid identity with their parent sequences. Power-law relationships were fitted, omitting points for any indels of size 3*n*, with *n *any positive integer. Expected ratios for insertions versus deletions were taken from this data; the expected number of indels per nucleotide substitution for several mammals was culled from the literature [21,22]. The program DNADIST [23] was used to calculate the nucleotide-level divergence of the PRs from ancestral sequences (calculated using PAML [20]; see section on Ka/Ks analysis above). This divergence value is used as a target in simulations. For each *PR*, repeated simulations of the evolution of the ancestral sequence towards present-day, for 1000 iterations, was performed using a Kimura two-parameter model. In each case, the resulting simulated protein coding sequence was marked for frame disablements (stop codons and frameshifts). *PR *sequences whose simulations yielded frame-disrupted sequences ≥ 99% of the time were labeled as having significant RFC.

### Assignment of functional categories

GO (Gene Ontology; [24]) functional categories were taken from the annotation files provided on the Ensembl [15] and Gene Ontology websites [25]. Further GO term annotations were derived by mapping functional GO annotations for the PDB (also downloaded from the GO website) onto Ensembl protein annotations, using 50% sequence identity and 0.8 fractional sequence coverage (for the protein domain) as thresholds, using alignment made by the program BLASTP (e-value ≤ 0.0001) [16]. These thresholds were benchmarked on the complete SCOP protein domain sequence database [26], to give a 2% false positive rate for GO term transfer.

### Mapping of cDNAs/mRNAs

Refseq mRNAs and complete Unigene consensus sequences were downloaded from the NCBI website [27], for both human and mouse. These were mapped to the coding sequences of Ensembl gene annotations, using blastn (e-value ≤ 1 × 10^-10 ^for alignments ≥ 100 nucleotides) [16]. All mappings that match with ≥ 99% sequence identity over ≥ 0.99 of the sequence length of the cDNA or mRNA, after removal of any polyadenylation, were retained. Further restriction of analysis of cDNA/mRNA mappings to those that do not match their putative parent sequences with >95% sequence identity, does not change the trends reported with regard to transcription evidence reported below.

## Results and discussion

The pipelines for annotating the complement of gene retrotranspositions (both *retropseudogenes *(*RψG*s) and putative *retrogenes *(*PRs*)) were applied to eight vertebrates. In particular, we focused on the mammals, to analyse the ages of putative retrogenes (*PRs*), to derive genome- and lineage-specific lists and to check for spurts of gene retrotransposition activity. We then examined for evidence of transcription (mRNA and cDNA mapping), involvement in alternative splicing, selection pressures (significant Ka/Ks values and reading-frame conservation), and for functional categorizations of parent genes.

### Overview of gene retrotranspositions in vertebrates

Our analysis suggests that up to ~3% of the genes encoded in a vertebrate genome contain a *PR *(Table 1), with the smallest percentages in the chicken and puffer fish *T. nigroviridis*. By comparison, mammalian genomes have ~2,000–5,000 retropseudogenes (*RψG*s), that have at least 70% of the coding sequence of their parent genes, again with smaller numbers in non-mammal vertebrates (just 644 *RψG*s in *T. nigroviridis*) (Table 1). These results together indicate that there has been less, recent gene-retrotransposition activity in the two non-mammal vertebrates. These observations tally well with other evidence for less retrotransposition activity in chicken and *Tetraodon*. In chicken, there appear to be little or no SINEs [28], and only ~8% of the genomic DNA is comprised of the CR1 ('chicken repeat 1') LINE-1 [28,29], whose reverse transcriptase is thought not to copy polyadenylated mRNAs [29]. In Tetraodon, <1% of the genome is comprised of retrotransposons, so gene retrotransposition should consequently be less likely [30]. For the eight genomes studied here, there are no significant linear correlations between the number of genes *or *proteins from a genome, versus the number of *PRs*, *or *RψGs (data not shown). Small percentages of the *PRs *could be classified as homologs of retrotransposed transposable elements, such as LINEs (2–12%), or as overlapping pseudogene annotations (4–13%).

As described in detail in *Methods*, we applied a 'local gene order' test, to set aside any *PRs *that may have arisen through local or segmental duplication, specifically for the human and mouse genomes (Table 1). This filter allows for at most one homologous protein encoded within a window of +/-3 genes along the genomic DNA (*i.e*., N_*homologs *_≤ 1) (Table 1). The substantial majority of human and mouse *PRs *pass this filter (80–87%).

### Ages of primate and rodent gene retrotranspositions

**Figure 4:**
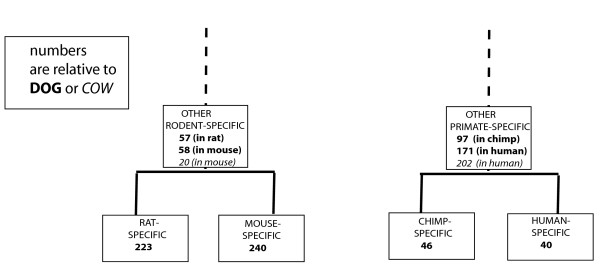
**Lineage-specific lists of PRs**: The number of species-specific PRs relative to other species. PRs specific relative to other species were obtained by comparison of *Ks *between the PR and its parent and the *Ks *between the parent (*Ks*_*PR_parent*_) and the ortholog of the parent in the other species (*Ks*_*parent_ortholog*_). PRs with *Ks*_*PR_parent *_<*Ks*_*parent_ortholog *_were defined as specific PRs relative to the other species. Only PRs which amino acid identity to parents is more than 70% and have an ortholog in other species were subjected to this calculation. Orthology criteria used are 40% identity over 60% length overlap. 'Human-specific' and 'Chimp-specific' PRs are those formed since the species diverged from each other; similarly, for 'Mouse-specific' and 'Rat-specific' PRs. 'Other primate-specific' are any other PRs formed in human or chimp since divergence from dog (in **bold **typeface), or from cow (in *italic *typeface); similarly, for 'Other rodent-specific'.

How old are these *PRs*? Is there any evidence for spurts of gene retrotransposition activity in mammalian evolution? To answer these questions, we examined the distribution of Ks values for *PRs *compared to their assigned parent genes, in the human and mouse genomes. (Only *PRs *passing the *local gene order *test, with threshold N_*homologs *_≤ 1 were analysed.) Ks is the rate of synonymous substitutions per synonymous site in codons, and has been generally used to age coding sequences. From comparing Ks values for *PRs*, their parents, and orthologs of their parents, we have also been able to derive lists of genome-specific and lineage-specific *PRs *(Figure 4; see *Methods *for details).

**Figure 3:**
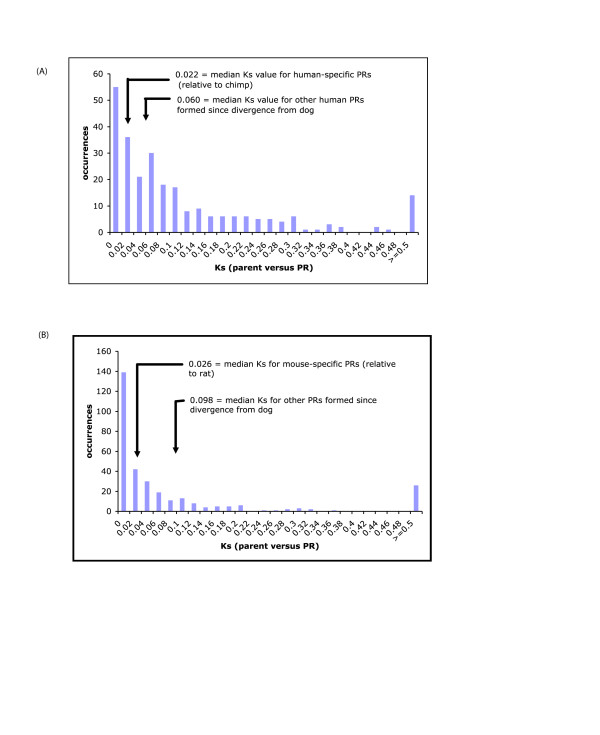
**Ks distributions: (A) **Ks distribution for human PRs meeting the local gene order test with threshold of N_*homologs *_= 0, from comparison to their parent sequences. Labelled are the median values for the 'Human-specific' set, and those PRs formed between divergence from dog and from chimp [see panel (C)]. A similar distribution is observed with an N_*homologs *_threshold of ≤ 1 for the local gene order test. **(B) **Ks distribution for mouse PRs meeting the local gene order test with threshold of N_*homologs *_= 0, from comparison to their parent sequences. Labelled are the median values for the 'Mouse-specific' set, and those PRs formed between divergence from dog and from chimp [see panel (C)]. A similar distribution is observed with an N_*homologs *_threshold of ≤ 1 for the local gene order test.

In human *PRs*, we see that there is a bimodal distribution of Ks (Figure 3A). The median Ks values for lists of *PRs *that are *human-specific *or that have otherwise been formed since divergence from dog, are labelled on the Ks histogram. The peak at Ks ~0.06–0.08 corresponds approximately to the median Ks value for *PRs *formed between human divergence from dog and from chimpanzee. This peak has been noted previously in analyses of retropseudogenes and total retrosequence populations [2,3,12], peaking at approximately the point of human lineage divergence from the New World Monkeys, some ~40 million years ago [3]. The peak at 0.0–0.02 (containing 21% of *PRs*) obviously corresponds to human-specific *PRs*. Some of these *PRs *may simply be too young to differentiate as a *PR *or retropseudogene (They have not been around long enough to acquire (apparent) reading-frame disablements.) Evidence for selection pressures on these sequences is discussed below.

By comparison, in the rodents, there is more, very recent gene retrotransposition activity. In mouse, we find proportionately more, genome-specific *PRs *(relative to rat), with 44% having Ks ≤ 0.02 (Figure 3B). In the two rodents, mouse and rat, there are >200 genome-specific *PRs*, compared to ~40 in each of the primates human and chimp. However, setting aside genome-specific examples, there are more gene retrotranspositions appearing in the primate lineage since its divergence from the dog or cow lineage (Figure 4).

These observations are in keeping with the apparent maintenance of greater levels of LINE and SINE retrotransposition activity in the rodents [31,32]; also, they tally well with previous observations for a general fall-off in such retrotransposition activity in the primate lineage [2,33].

### Transcription evidence

Focussing on human and mouse, we examined the proportion of *PRs *that could be mapped to a complete Unigene consensus cDNA or a complete Refseq mRNA from the NCBI ([27]; see *Methods *for details). For both organisms, we found that the *PRs *have significantly less mapping of this transcription evidence (P < 0.001, using the z-score for distribution of the sample mean). For human, only 23% of human *PRs *mapped to a Refseq mRNA or Unigene cDNA consensus sequence (compared to 41% for the whole proteome). This may be due to lower transcription levels, because they are novel gene sequences using co-opted promoter elements at a site distal to the genomic location of their parent genes [11]. This general reduction in transcription is also to be expected, if some of the sequences are recent pseudogenes without disablements.

In addition, we examined how many *PRs *arise from alternatively-spliced genes. To do this, we cross-referenced the PR data with alternative splicings classified in the Alternative Splicing Database (ASD) at the EBI [34]. We found that 24% of genes for human PRs arose from an alternatively spliced gene, compared to 59% of genes overall (significantly less, P < 0.001 using the z-score for distribution of the sample mean). A significant reduction in representation from alternatively-spliced genes was also observed in mouse (4%, compared to 29% overall).

**Figure 5:**
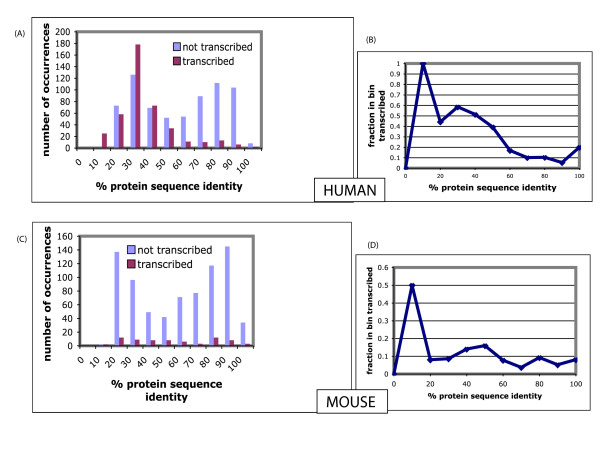
**Distributions of percentage protein sequence identity between PRs and parents**. **(A) **Distribution of % protein sequence identity for all human PRs that pass the local gene order test (N_*homologs *_≤ 1). These are broken down into 'transcribed' and 'not transcribed'. **(B) **The fraction that are transcribed in each bin of the histogram in panel A. **(C) **Distribution of % protein sequence identity for all mouse PRs that pass the local gene order test (N_*homologs *_≤ 1). These are broken down into 'transcribed' and 'not transcribed'. **(D) **The fraction that are transcribed in each bin of the histogram in panel C.

We examined the divergence of *PRs *from their putative parents, in the context of transcription evidence. This is illustrated in Figure 5 for those *PRs *that pass the local gene order test for both mouse and human, with N_*homologs *_≤ 1. In human, there is a marked difference in the behaviour of transcribed *PRs *(purple bars in Figure 5A), compared to those without transcription evidence (blue bars in Figure 5A). There are relatively very few transcribed *PRs *with high sequence identities (*i.e*., that formed relatively recently). The bimodal character of these plots may arise because some of the PRs: (i) are in a younger population of *PRs *that are not under selection pressures, but which have not accumulated deleterious mutations, simply by chance; *i.e*., they are pseudogenes without disablements; *or *(ii) are in a state of relaxed selection, and thus concomitantly have low transcription levels. Similarly, only a small fraction of the *PRs *calculated to have formed since divergence from the dog lineage (Figure 4), in either human (15/207, 7%) or mouse (20/292, 7%), are transcribed (significantly less, P < 0.05 using χ^2 ^tests for both cases).

### Ka/Ks and reading frame conservation (RFC) analysis

Is there any evidence for selection pressures on the *PRs *in human and mouse? We investigated this question for *PRs *that have been formed in human and mouse, since their divergences from dog. One standard indicator of selection pressures is the Ka/Ks ratio. This is the number of non-synonymous mutations per non-synonymous site, over the number of synonymous mutations per synonymous site, in codons. Negative (or 'purifying') selection in a specific lineage is indicated by a value significantly <1.0, where positive ('diversifying') selection is demonstrated by a value significantly >1.0. We calculated Ka/Ks values for *PRs *relative to ancestral sequences for the parents of the *PRs *(see *Methods *for details). We tested whether any of these Ka/Ks values were significantly < or >1.0 by generating 500 random pairs of sequences as diverged as the PR and ancestral sequence (to calculate expected means and standard deviations for the Ka/Ks values), and then deriving a P-value for the observed Ka/Ks.

Strikingly, when we correct for multiple hypothesis testing in the Ka/Ks calculations, we find only one *PR *sequence (formed since divergence from dog) that is under significant selection at the codon level in the human genome, and none in the mouse genome. (The one significant human example is a PR under purifying selection, from a family of proteins with the GTP-binding SAR1 domain.)

**Figure 6:**
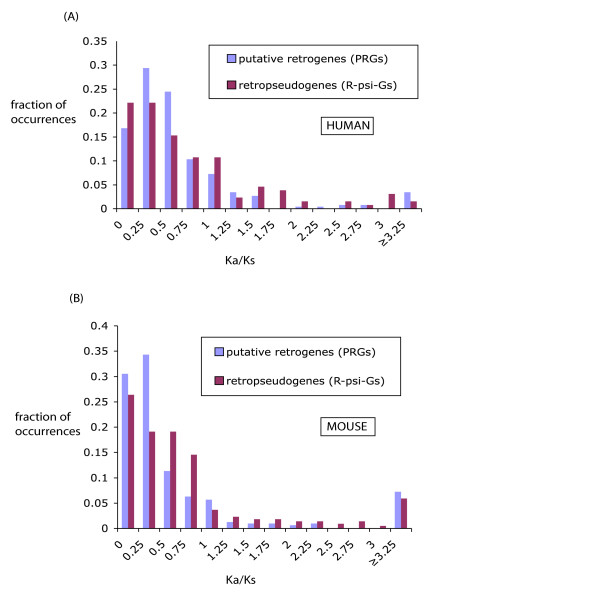
**Ka/Ks distributions for PRs and for retropseudogenes (*RψG *s)**. **(A) **Distribution of Ka/Ks for human PRs (n = 262) meeting the local gene order test (N_*homologs *_≤ 1), compared to Ka/Ks for the *RψG*s (n = 183). All sequences were required to have protein sequence identity ≥ 60.0% with their parent sequences. **(B) **As in (A), but for mouse PRs (n = 318) and *RψG*s (n = 220).

In addition, we calculated the distribution of Ka/Ks values from directly comparing the *PRs versus *their parents. From this specific sort of comparison, the neutral expectation for Ka/Ks is not ~1, because of non-synonymous mutations accumulating in the parent genes [2]. A significant excess of Ka/Ks values <0.5, however, may be indicative of purifying selection in the data set. For comparison, we also similarly calculated a Ka/Ks distribution for *RψG*s *versus *their respective parents, carefully parsing out disablements (frameshifts and premature stop codons) from the *RψG *sequences. This Ka/Ks distributional analysis is performed for both human and mouse (Figure 6). For human (Figure 6A), we find no significant excess of *PRs *with Ka/Ks values <0.5 relative to *RψG*s, either for the whole data set of *PRs*, or for the subset formed in the primate lineage, contrary to a previous report [12] (χ^2 ^test or Fisher's exact test). This distribution is thus consistent with a set of largely neutral retrotranspositions, behaving like *RψG*s. However, for mouse-specific *PRs*, there is a significant excess with Ka/Ks <0.5 (P ≤ 0.001, χ^2 ^test and Fisher's exact test) (Figure 6B), indicating that some of these mouse *PR *sequences are under purifying selection.

Conservation of open reading frames without disablements (frameshifts or stop codons), can also be an indicator of coding ability [12,35,36]. We derived a method for assessing significant conservation of open reading frames, using simulation with power-law insertion/deletion (indel) statistics [4]. Using simulations with calculated neutral rates of substitution, insertion and deletion, the likelihood of conservation of an open reading frame without interruption by frameshifts and stop codons, can be determined (see *Methods *for details). To give sufficient power, a P-value threshold of ≤ 0.01 was used as an indicator for significant reading-frame conservation (RFC). This calculation is complementary to the Ka/Ks analysis.

The results are listed Table 2. Even though there were no significant Ka/Ks values in mouse, we find over 30 mouse-specific *PRs *that have significant reading frame conservation, and a further 17 that were formed since divergence from dog (Table 2). In human, we find in total, 59 *PRs *with significant RFC, that have arisen since divergence from dog (Table 2). A phylogenetic tree for an example of one of the mouse PRs with significant RFC, which is homologous to citrate synthase, is depicted in Additional Figure 3, with a depiction of the chromosomal milieu of this PR and of its parent in Additional Figure 4. Two further representative examples of human PRs are also shown in Additional Figure 4 (one with significant RFC and the other without), with varying degrees of age and transcription evidence.

**Table 2:** Results of analysis for reading-frame conservation (RFC)

**Species**	**Set †**	**Number with significantly conserved reading frame ††**
**Human**	Human-specific relative to chimp	10/40 (25%)
	Others in human, that were formed since divergence from dog	49/171 (29%)
	Other older PRs	162/378 (43%)
	TOTAL	221/589 (38%)
**Mouse**	Mouse-specific relative to rat	35/240 (15%)
	Others in mouse, that were formed since divergence from dog	17/58 (29%)
	Other older PRs	123/233 (53%)
	TOTAL	175/531 (33%)

† These are the sets illustrated in Figure 4. All of the PRs that pass the local gene order test, allowing for N_*homologs *_≤ 1 were analysed for reading-frame conservation.

†† For PRs formed since divergence from dog, RFC simulations were performed using ancestral sequences calculated using PAML [20]. For PRs formed before divergence from dog, RFC simulations were conservatively performed to half of the nucleotide-level divergence between the PR and its assigned parent sequence.

These results are evidence for conservation of protein open reading frames, even though we found no evidence for purifying selection from examination of the sequences individually for Ka/Ks. This would arise if the *PRs *were generally under relaxed or positive selection pressures at the codon level. The existence of relaxed selection is consistent with the markedly low numbers of *PRs *found to be transcribed in both human and mouse, particularly those that were formed since divergence from dog.

Out of those with significant RFC, is there any evidence for non-neutral Ka/Ks trends? We checked for significant excess of *PRs *with Ka/Ks values <0.5, > 0.5, <1.0 or >1.0 for each of the subsets listed (Table 2). In the human lineage, we find a significant excess of *PRs *with Ka/Ks >0.5 (40/59, P < 0.05, χ^2 ^test and Fisher exact test, compared to an expectation from *RψG *sequences) formed between divergence from dog and from chimp. This non-neutral trend is suggestive of positive selection distributed throughout this specific subset population of *RψG *sequences. Out of the other subsets listed in Table 2, the only other significant non-neutral Ka/Ks tendency is for an excess of mouse-specific *PRs *to be under purifying selection (26/35 having Ka/Ks <0.5, compared to an expectation from *RψG *sequences, P < 0.05, χ^2 ^test and Fisher exact test).

### Functional categories

To assess whether the *PRs *and *RψG*s have any unusual functional associations, we assigned functional categories using the Gene Ontology (GO) functional classification (Table 3). As noted previously, '*Structural constituent of ribosome*' is a prevalent functional category for *RψG*s [2,3,14]. Noticeably, for mouse, there are more retropseudogenes in metabolic categories, than in human (Table 3). A notable prevalence indicative of origin in retrotransposition, '*structural constituent of ribosome*', occurs in the top ten of all *PR *(sub)sets, and is ranked number one for *PRs *formed since divergence from dog, for both mouse and human (Table 3). *'Structural constituent of ribosome' *is also the only Gene Ontology term that is statistically overrepresented in all of the retrotransposed gene sets listed (Table 3 legend; *P' *<0.05, using binomial statistics and a Bonferroni correction for multiple hypothesis testing [37]). The functional category preferences are not caused by over-representation of any one parent, since when representations of *PR*s on a parent-by-parent basis are tallied up, we find only a very small number of parents giving rise to five or more *PR*s (Suppl. Table 1); the substantial majority of parents have only one PR offspring (286/353 [81%] for mouse, and 279/347 [80%] for human).

**Table 3:** Most common Gene Ontology (GO) functional terms for different sets of sequences *

**Human**			
**All genes (Total = 33930)**	**retropseudogenes (Total = 2493)**	**All PRs (Total = 631)**	PRs formed since divergence from dog lineage (Total = 211) **

GO:0005515, protein binding (2360)	***GO:0003735, structural constituent of ribosome (203) ***	GO:0008270, zinc ion binding (49)	***GO:0003735, structural constituent of ribosome (11)***
GO:0008270, zinc-ion binding (2069)	GO:0008270, zinc ion binding (189)	GO:0006355, regulation of transcription, DNA-dependent (35)	GO:0003677, DNA binding (10)
GO:0006355, regulation of transcription, DNA-dependent (2029)	GO:0006355, regulation of transcription, DNA-dependent (166)	GO:0005509, calcium ion binding (25)	GO:0006355, regulation of transcription, DNA-dependent (9)
GO:0005524, ATP-binding (1687)	**GO:0003676, nucleic acid binding (132)**	GO:0005525, GTP binding (21)	GO:0005525, GTP binding (5)
GO:0003677, DNA binding (1339)	**GO:0003723, RNA binding (126)**	GO:0005515, protein binding (21)	GO:0003823, antigen binding (5)
GO:0007165, signal transduction (1264)	GO:0005515, protein binding (114)	GO:0004842, ubiquitin-protein ligase activity (21)	GO:0003676, nucleic acid binding (5)
GO:0016740, transferase activity (1263)	GO:0003677, DNA binding (110)	GO:0003677, DNA binding (20)	GO:0030145, manganese ion binding (4)
GO:0004872, receptor activity (1242)	GO:0005524, ATP binding (93)	GO:0003676, nucleic acid binding (20)	GO:0020037, heme binding (4)
GO:0016787, hydrolase activity (1171)	GO:0046872, metal ion binding (63)	***GO:0003735, structural component of the ribosome (16) ***	GO:0016757, transferase activity, transferring glycosyl groups (4)
GO:0003700, transcription factor activity (1052)	GO:0000166, nucleotide binding (57)	GO:0003723, RNA binding (13)	GO:0005509, calcium ion binding (4)

**Mouse**			

**All genes (Total = 32442)**	**Retropseudogenes (Total = 2969)**	***PRs *(Total = 663)**	*PRs *formed since divergence from dog lineage (Total = 298) **

GO:0005515, protein binding (2502)	**GO:0003676, nucleic acid binding (273)**	GO:0005515, protein-binding (17)	***GO:0003735, structural constituent of ribosome (16) ***
GO:0004872, receptor activity (1923)	**GO:0051287, NAD binding (243)**	*GO:0003735, structural constituent of ribosome (16)*	GO:0005524, ATP binding (8)
GO:0006355, regulation of transcription, DNA-dependent (1571)	**GO:0008943, glyceraldehyde-3-phosphate dehydrogenase activity (243)**	GO:0008270, zinc ion binding (12)	GO:0005515, protein-binding (7)
GO:0008270, zinc ion binding (1481)	**GO:0004365, glyceraldehyde-3-phosphate dehydrogenase (phosphorylating) activity (243)**	GO:0006355, regulation of transcription, DNA-dependent (12)	GO:0016740, transferase activity (6)
GO:0005524, ATP binding (1252)	**GO:0008270, zinc ion binding (235)**	GO:0005524, ATP binding (12)	GO:0016491, oxidoreductase activity (6)
GO:0016740, transferase activity (1036)	***GO:0003735, structural constituent of ribosome (201) ***	GO:0005509, calcium ion binding (12)	GO:0006355, regulation of transcription, DNA-dependent (6)
GO:0003677, DNA binding (1017)	GO:0005515, protein-binding (101)	GO:0016740, transferase activity (10)	GO:0016853, isomerase activity (5)
GO:0016787, hydrolase activity (911)	**GO:0016491, oxidoreductase activity (94)**	GO:0016787, hydrolase activity (9)	GO:0016787, hydrolase activity (5)
GO:0000166, nucleotide binding (873)	GO:0005524, ATP binding (78)	GO:0003677, DNA binding (9)	GO:0003677, DNA binding (5)
GO:0003676, nucleic acid binding (872)	**GO:0004190, aspartic-type endopeptidase activity (77)**	GO:0003676, nucleic acid binding (9)	GO:0016874, ligase activity (3)

* The most abundant Gene Ontology 'molecular function' terms are listed for each set of sequences, in decreasing order of abundance. The GO term number and a brief description are followed by the number of occurrences (in brackets). Significant overrepresentation of GO terms was calculated as described previously using binomial statistics, using a Bonferroni correction for multiple hypothesis testing (*P' *< 0.05) [37]. '*Structural constituent of the ribosome*' (in italics) is the only term that is significantly overrepresented in all of the three putatively retrotransposed sequences.

** '*Structural constituent of the ribosome*' remains the most abundant GO category in this column when PRs from parents with large exons (FLE>0.67) are removed, or a more stringent N_homologs _threshold of = 0 is used.

## Conclusion

We have developed two parallel pipelines for the annotation of putative retrogenes (PRs) and retropseudogenes (RψG) in whole genomes. The new pipeline for retropseudogene annotation employs length-based heuristics to speed up the processing of sequence alignment data. We used these pipelines on the vertebrates here, but they are readily applicable to any genome and its set of gene/protein annotations. Genome analysis is constantly in flux, and so obviously, as vertebrate genome assemblies and their annotations are streamlined further, we will be further able to refine our retrotransposition analyses, to remove any errors from missing gene annotations, small genome assembly gaps, *etc*.

We focussed on the annotation of retro(pseudo)genes in mouse and human. We were particularly interested in the retro(pseudo)genes formed since divergence from an 'outgroup' genome, that of the dog. We found evidence for excess, recent gene-retrotransposition activity in both human and mouse, since their divergences from the dog lineage. We find some evidence for selection on PRs at different phases of mouse and human genome evolution. In human, there is statistical evidence for non-neutral evolution (suggestive of positive selection), for population of PRs that have significantly conserved reading frames and that formed since divergence from the dog lineage. Also, we found that, human PRs formed since divergence from the dog lineage have significantly less transcription evidence, which is consistent with the possibility that they are pseudogenes, or in some intermediate phase of relaxed selection. Such a state of low expression coupled with relaxed selection may also arise for alternatively-spliced exons [38,39]. In summary, our genomic analysis suggests that some human PRs, formed since divergence from the dog lineage, are undergoing a form of non-neutral evolution, but the majority are either young pseudogenes (that are undisabled simply by chance), or lowly-expressed coding sequences in a state of 'relaxed' selection.

Further information on the PRs and RψGs is available at the website: [40].

## Abbreviations

PR, putative retrotransposition; *RψG*, retropseudogene; RFC, reading frame conservation; TE, transposable element; GO, Gene Ontology; PDB, Protein Data Bank; SCOP, Structural Classification of Proteins; LINE, Long Interspersed Element; SINE, Short Interspersed Element; FLE, fraction of largest exon.

## Authors' contributions

ZY developed the pipelines, performed most of the data analysis, and wrote initial drafts of the manuscript. DM performed some data analysis of alternative splicing events. MI performed phylogenetic analysis. PH conceived of the project, performed evolutionary analyses, and wrote later drafts of the manuscript.

## Supplementary Material

Additional file 1 Additional Figure 1: Distributions of the fraction of the largest exon (FLE). The fraction of the parent sequences that are taken up by their largest exons (denoted FLE) is plotted for various sets of sequences. Click here for file

Additional file 2 Additional Figure 2: Overlap of the three methods for determining species-specific or lineage-specific lists of PRs, for Human (top panel) and Mouse (bottom panel). Three different methods for determining the relative age of sequences were used for generating species-specific lists. This figure demonstrates the overlap between these methods. Click here for file

Additional file 3 Additional Figure 3: Example of a PR of citrate synthase in Mouse. A phylogenetic analysis was performed of citrate synthase PR from mouse. Click here for file

Additional file 4 **Additional **Figure 4: **Chromosomal milieus for three PR examples**. Screenshots taken from the Ensembl database, depicting nearby features for three PRs and their putative parents. Click here for file

Additional file 5 Parents with most PRs. The parent genes that spawn most PRs are listed. Click here for file
