# Supplementary material for: Analysis of the role of retrotransposition in gene evolution in vertebrates
Source: BMC Bioinformatics. 2007 Aug 24;8:308. doi: 10.1186/1471-2105-8-308 (PMC2048973; doi:10.1186/1471-2105-8-308)
Supplement: Additional file 1 — Additional Figure 1: Distributions of the fraction of the largest exon (FLE). The fraction of the parent sequences that are taken up by their largest exons (denoted FLE) is plotted for various sets of sequences. [file 1471-2105-8-308-S1.pdf]

(A)

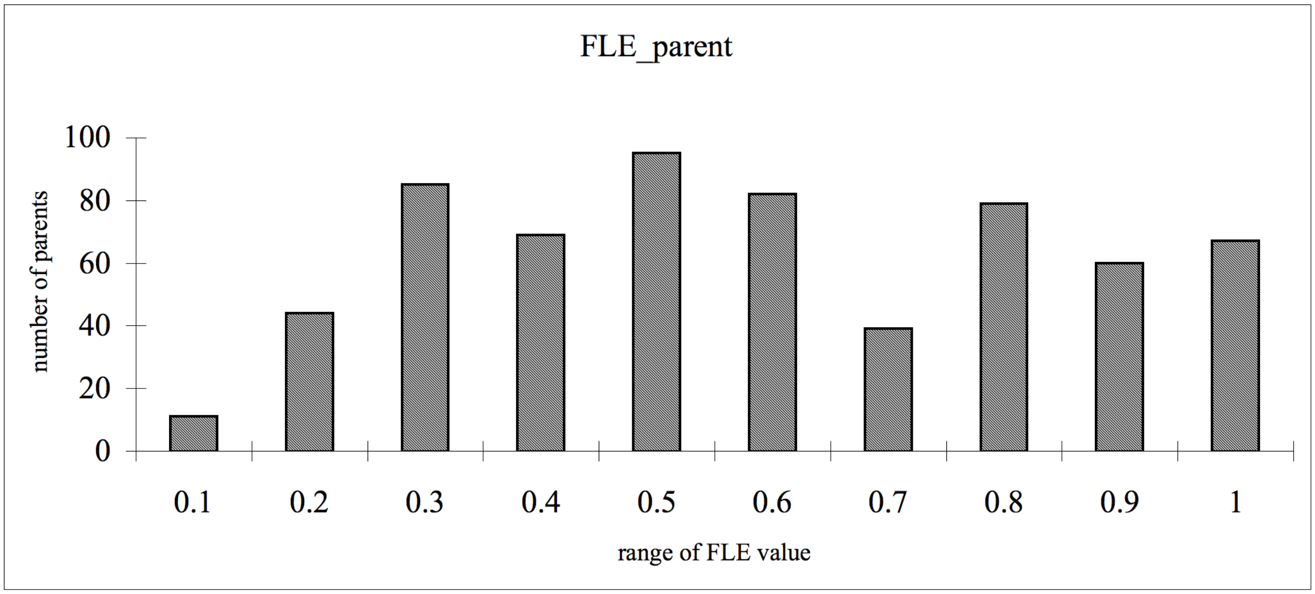
FLE_parent
100
80
60
40
20
0
number of parents
0.1
0.2
0.3
0.4
0.5
0.6
0.7
0.8
0.9
1
range of FLE value

(B)

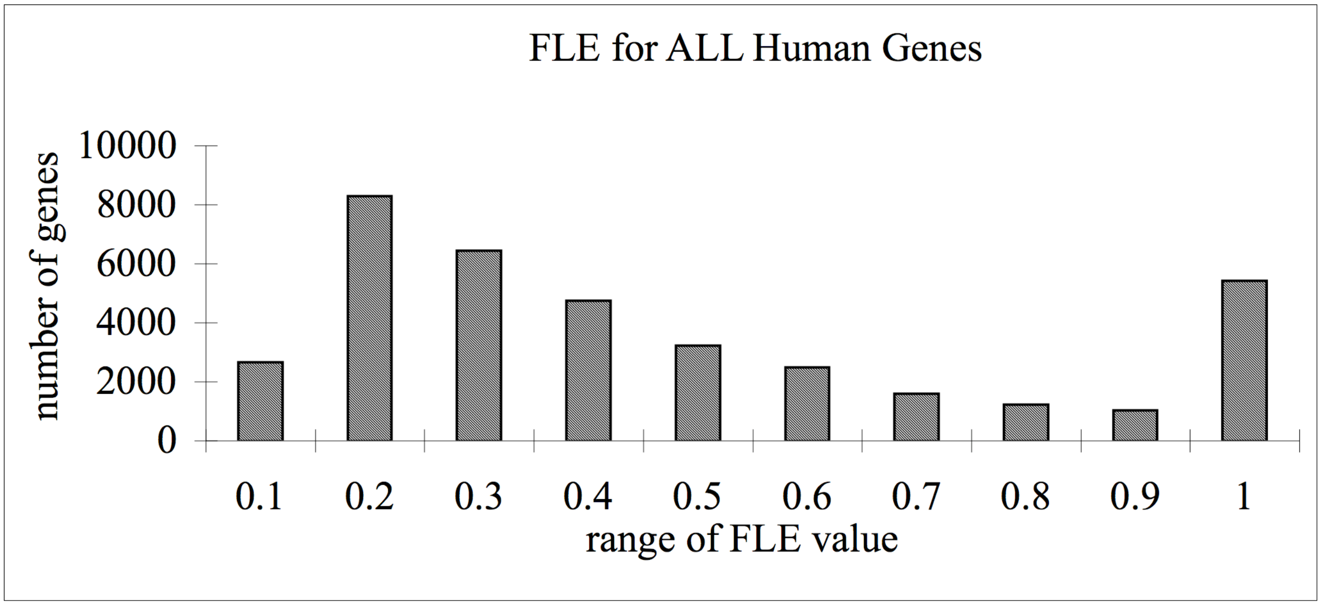
FLE for ALL Human Genes
10000
8000
6000
4000
2000
0
number of genes
0.1
0.2
0.3
0.4
0.5
0.6
0.7
0.8
0.9
1
range of FLE value

(C)

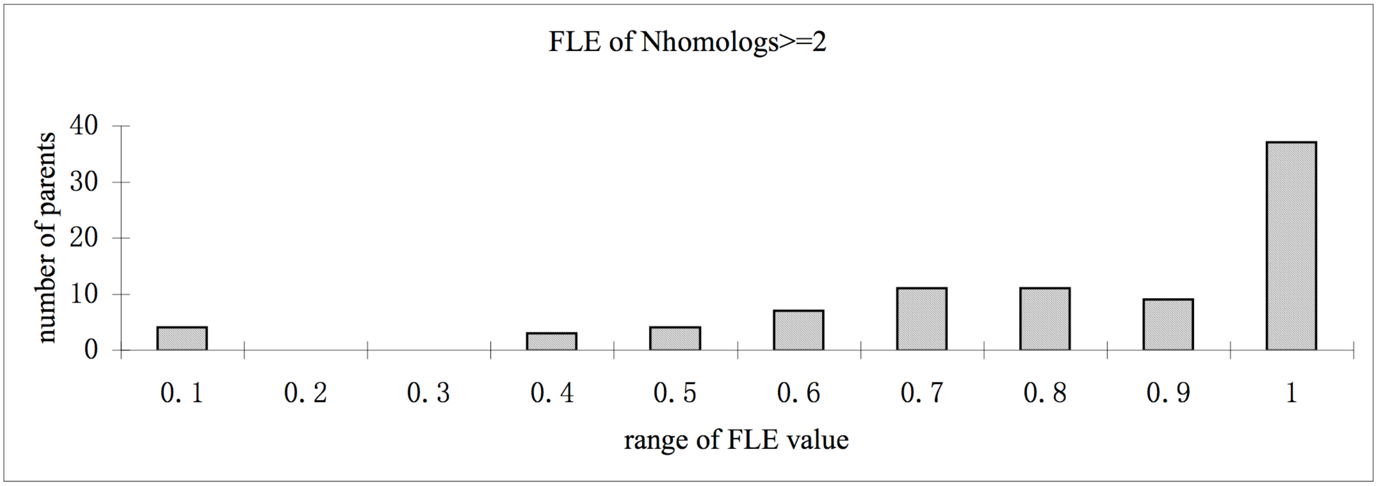

(D)

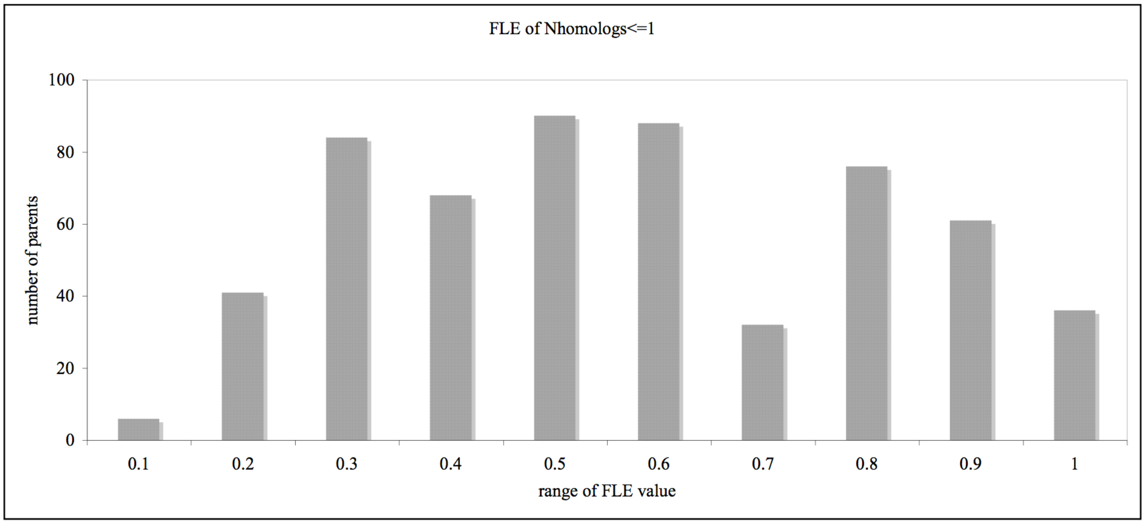

Supplementary Figure 1: Distributions of the fraction of the largest exon (FLE). FLE is the 'fraction of the largest exon', covered within the coding sequence of the proteins. We derived histograms for this measure for variouse sets of sequences. Histogram bins labelled *x* contain all values such that *x*-0.1 <

FLE ≤*x*. **(A)** We calculated FLE_{parent} for all parent genes for human PRs. FLE_{parent} has an approximately uniform distribution for this data set. **(B)** The overall distribution for human genes shows a peak in FLE between 0.2-0.3; the peak at 0.9-1.0 is due to single-exon genes. **(C)** The distribution of FLE_{parent} for all human PRs with $N_{homologs} \geq 2$, according to the local gene order test (see *Methods* for details). Note that the putative parents for these PRs tend to have a single large exon; more than two-thirds of these apparent PRs arise from genomic clusters of Zn-finger containing proteins. **(D)** As for (C), but with $N_{homologs} \leq 1$.
