# Supplementary material for: Analysis of the role of retrotransposition in gene evolution in vertebrates
Source: BMC Bioinformatics. 2007 Aug 24;8:308. doi: 10.1186/1471-2105-8-308 (PMC2048973; doi:10.1186/1471-2105-8-308)
Supplement: Additional file 2 — Additional Figure 2: Overlap of the three methods for determining species-specific or lineage-specific lists of PRs, for Human (top panel) and Mouse (bottom panel). Three different methods for determining the relative age of sequences were used for generating species-specific lists. This figure demonstrates the overlap between these methods. [file 1471-2105-8-308-S2.pdf]

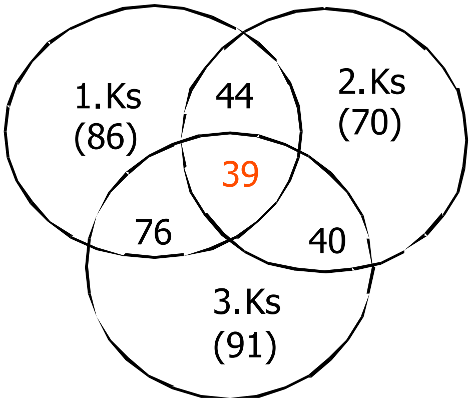

Human specific PR relative to Chimp

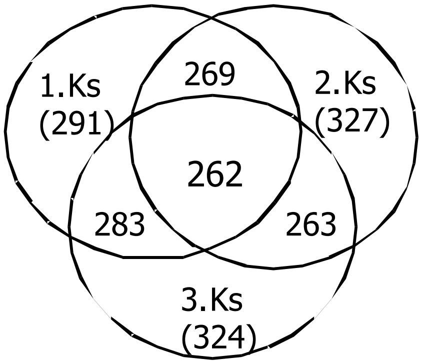

Mouse specific PR relative to Rat

Supplementary Figure 2: Overlap of the three methods for determining species-specific or lineage-specific lists of PRs, for Human (top panel) and Mouse (bottom panel). The three methods are numbered as in the main text

Methods section "*Analysis of K_s and K_a/K_s values, and derivation of genome- and lineage-specific gene lists*". The output of the three methods has high agreement for the generation of a Mouse-specific list. The intersection of all three sets occupies about 90 percent of method (1)'s output, 80 percent of method (2)s output and 81 percent of method (3)'s output. While for the generation of a Human-specific list, the intersection of all three sets overlap each output by the three methods at 45 percent, 56 percent and 43 percent respectively. The output of method (1) has very high overlap with output of method (3) in both Human-specific (the interaction has potion of 88% and 84%) and Mouse-specific (the interaction has potion of 88% and 84%). We used both method (3) for further calculation.
