# Supplementary material for: Analysis of the role of retrotransposition in gene evolution in vertebrates
Source: BMC Bioinformatics. 2007 Aug 24;8:308. doi: 10.1186/1471-2105-8-308 (PMC2048973; doi:10.1186/1471-2105-8-308)
Supplement: Additional file 3 — Additional Figure 3: Example of a PR of citrate synthase in Mouse. A phylogenetic analysis was performed of citrate synthase PR from mouse. [file 1471-2105-8-308-S3.pdf]

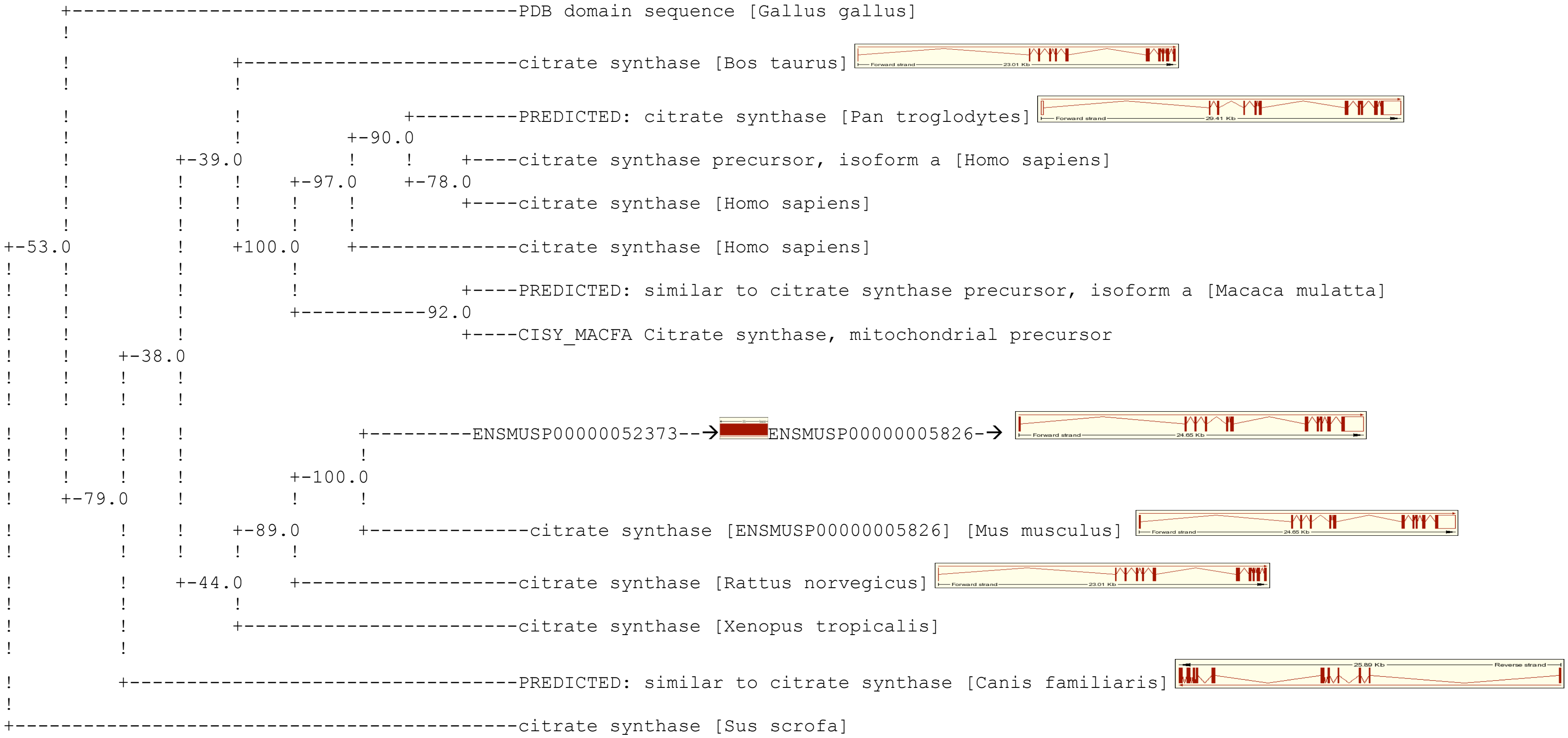

Supplementary Figure 3: Example of a PR of citrate synthase in Mouse. ENSMUSP00000052373 is a PR made from ENSMUSP00000005826, which is conserved in other mammals with the same intron-exon structure. Pictures of intron-exon structure were taken from the Ensembl website [http://www.ensembl.org]. The tree is a maximum-likelihood majority-rule consensus tree, that was made using PHYLIP v3.6b, based on CLUSTALW multiple alignments [ref.] of homologous proteins to ENSMUSP00000052373 in other species, that were collated using *blastp* [e-value $\leq 10^{-4}$] using the NCBI blast server and the *nr*

database [http://www.ncbi.nlm.nih.gov/BLAST/]. One hundred bootstrap replicates were generated using the PHYLIP *bootseq*, *proml* and *consense* programs [ref.].
