# Supplementary material for: Analysis of the role of retrotransposition in gene evolution in vertebrates
Source: BMC Bioinformatics. 2007 Aug 24;8:308. doi: 10.1186/1471-2105-8-308 (PMC2048973; doi:10.1186/1471-2105-8-308)
Supplement: Additional file 4 — Additional Figure 4: Chromosomal milieus for three PR examples. Screenshots taken from the Ensembl database, depicting nearby features for three PRs and their putative parents. [file 1471-2105-8-308-S4.pdf]

Supplementary Figure 4

*(a) Citrate synthase from Mouse (*ENSMUSP00000052373, *as in Suppl. Fig. 3)*

PR (ENSMUSP00000052373)
[P_{RFC} ≤0.01 ; Ka/Ks (PR-parent) = 0.21; Ka = 0.04; Ks = 0.19; no match to full-length cDNA]

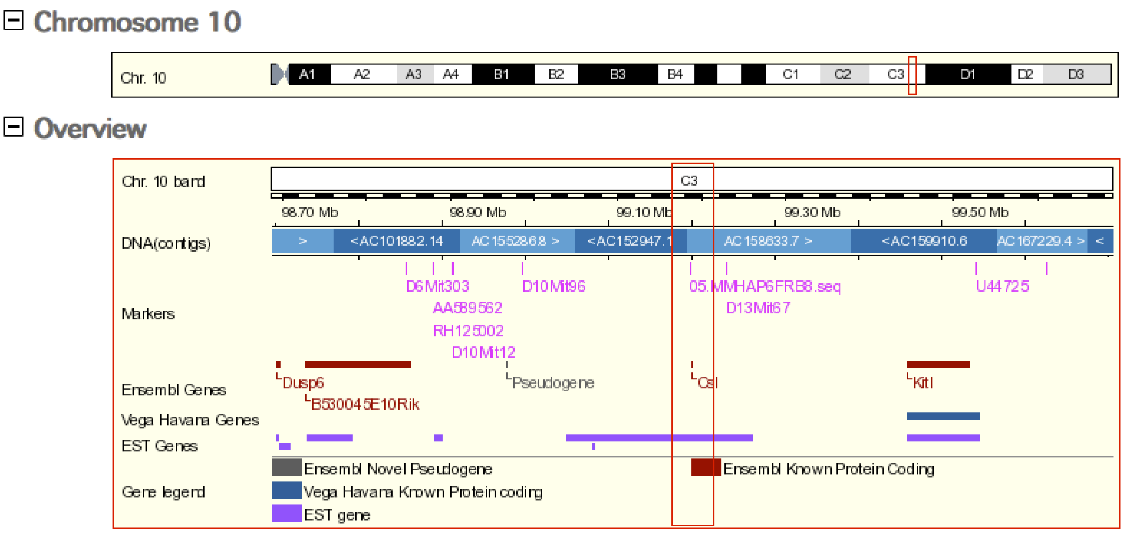

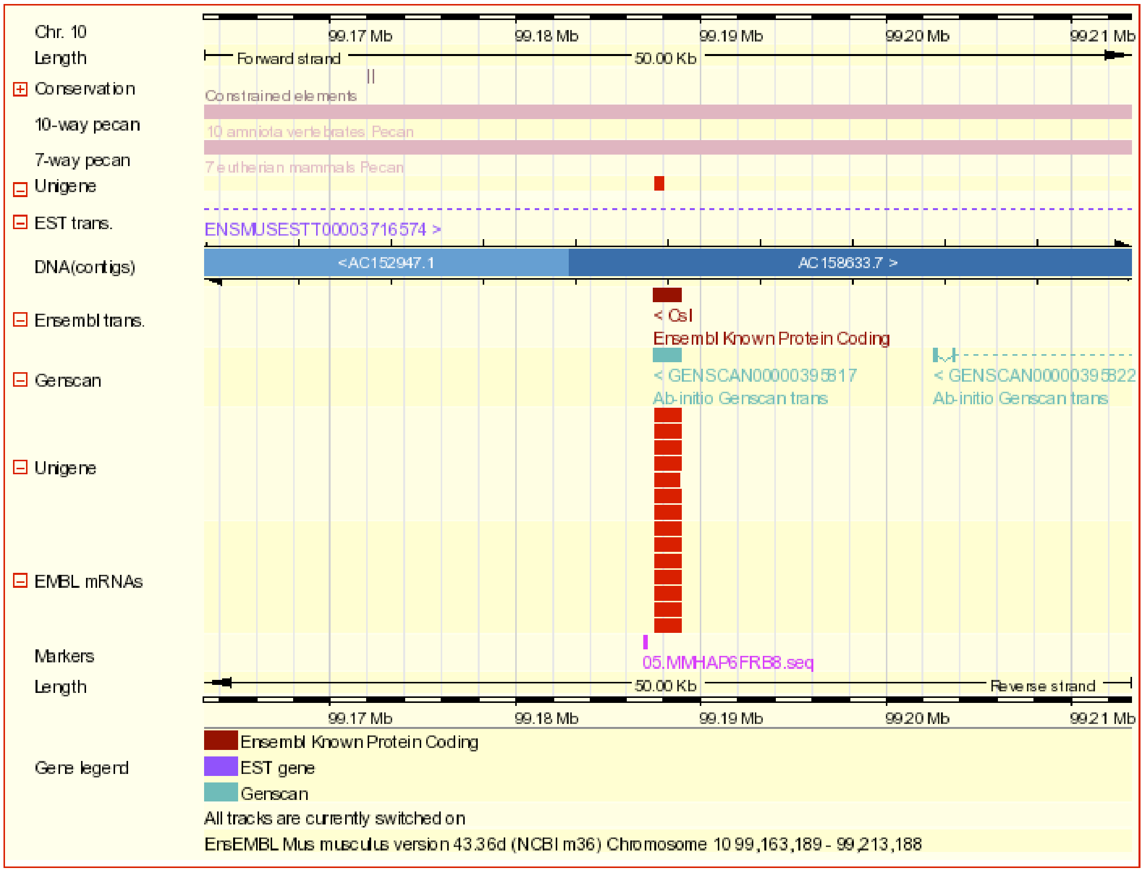

Parent (ENSMUSP00000005826)

Chromosome 10

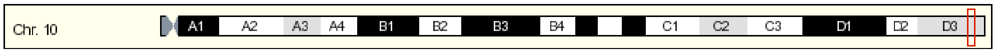

Overview

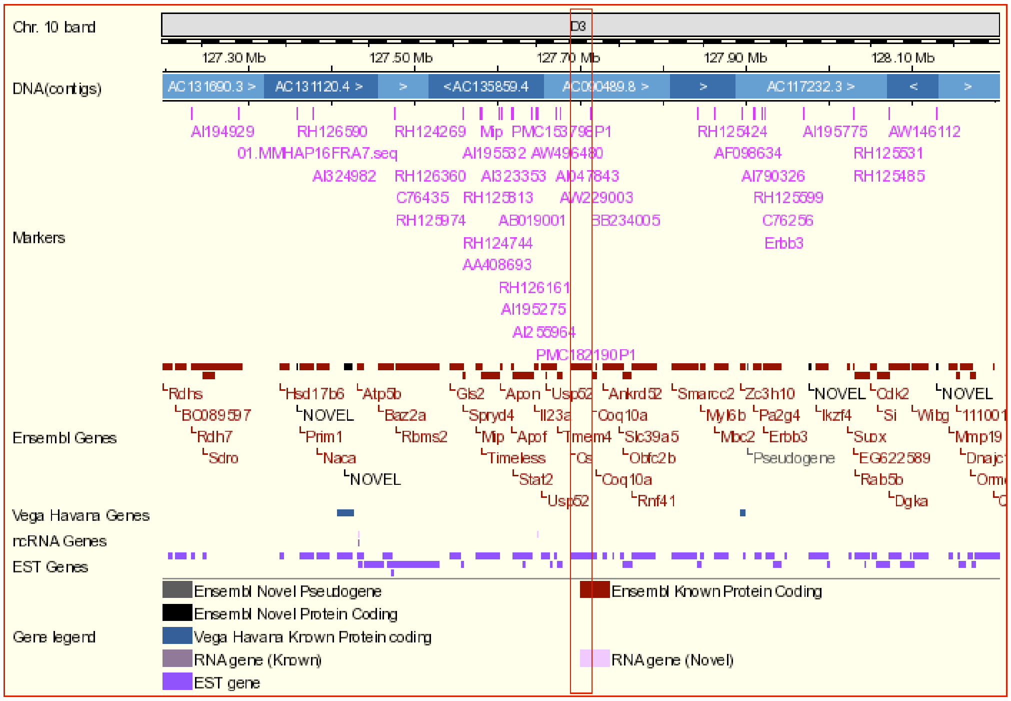

Chr. 10 band
D3
127.30 Mb
127.50 Mb
127.70 Mb
127.90 Mb
128.10 Mb
DNA(contigs)
AC131690.3 >
AC131120.4 >
<AC135859.4
AC090489.8 >
AC117232.3 >
Markers
AI194929
RH126590
RH124269
Mip
PMC153796P1
RH125424
AI195775
AW146112
01.MMHAP16FRA7.seq
AI195532
AW496480
AF098634
RH125531
AI324982
RH126360
AI323353
AI047843
AI790326
RH125485
C76435
RH125813
AW229003
RH125599
RH125974
AB019001
BB234005
C76256
RH124744
Erbb3
AA408693
RH126161
AI195275
AI255964
PMC182190P1
Ensembl Genes
Rdhs
Hsd17b6
Atp5b
Gls2
Apon
Usp52
Ankrd52
Smarcc2
Zc3h10
NOVEL
Cdk2
NOVEL
BC089597
NOVEL
Baz2a
Spryd4
Il23a
Coq10a
Myl6b
Pa2g4
Ikzf4
Si
Wibg
Rdh7
Prim1
Rbms2
Mip
Apof
Tmem4
Slc39a5
Mbc2
Erbb3
Suox
Mmp19
Sdro
Naca
Timeless
Cs
Obfc2b
Pseudogene
EG622589
NOVEL
Stat2
Coq10a
Rab5b
Usp52
Rnf41
Dgka
Vega Havana Genes
ncRNA Genes
EST Genes
Gene legend
Ensembl Novel Pseudogene
Ensembl Known Protein Coding
Ensembl Novel Protein Coding
Vega Havana Known Protein coding
RNA gene (Known)
RNA gene (Novel)
EST gene

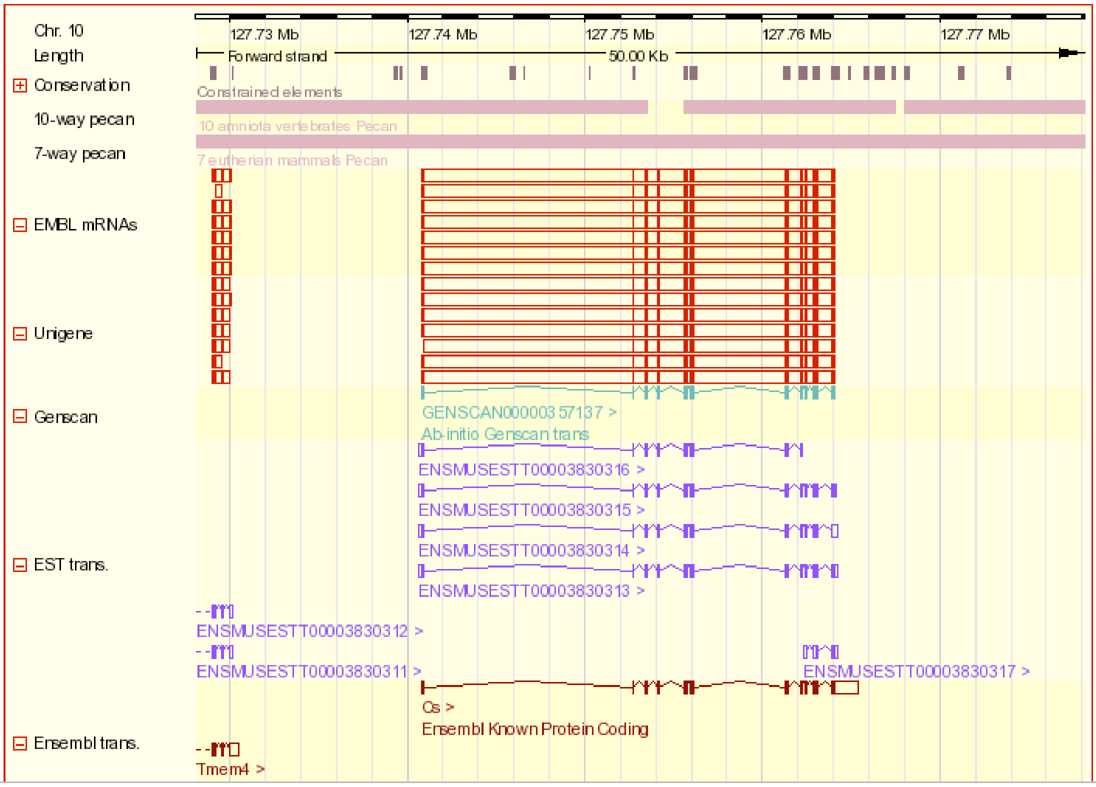

Chr. 10
127.73 Mb
127.74 Mb
127.75 Mb
127.76 Mb
127.77 Mb
Length
Forward strand
50.00 Kb
Conservation
Constrained elements
10-way pecan
10 amniota vertebrates Pecan
7-way pecan
7 eutherian mammals Pecan
EMBL mRNAs
Unigene
Genscan
GENSCAN00000357137 >
Ab-initio Genscan trans
EST trans.
ENSMUSESTT00003830316 >
ENSMUSESTT00003830315 >
ENSMUSESTT00003830314 >
ENSMUSESTT00003830313 >
ENSMUSESTT00003830312 >
ENSMUSESTT00003830311 >
ENSMUSESTT00003830317 >
Ensembl trans.
Cs >
Ensembl Known Protein Coding
Tmem4 >

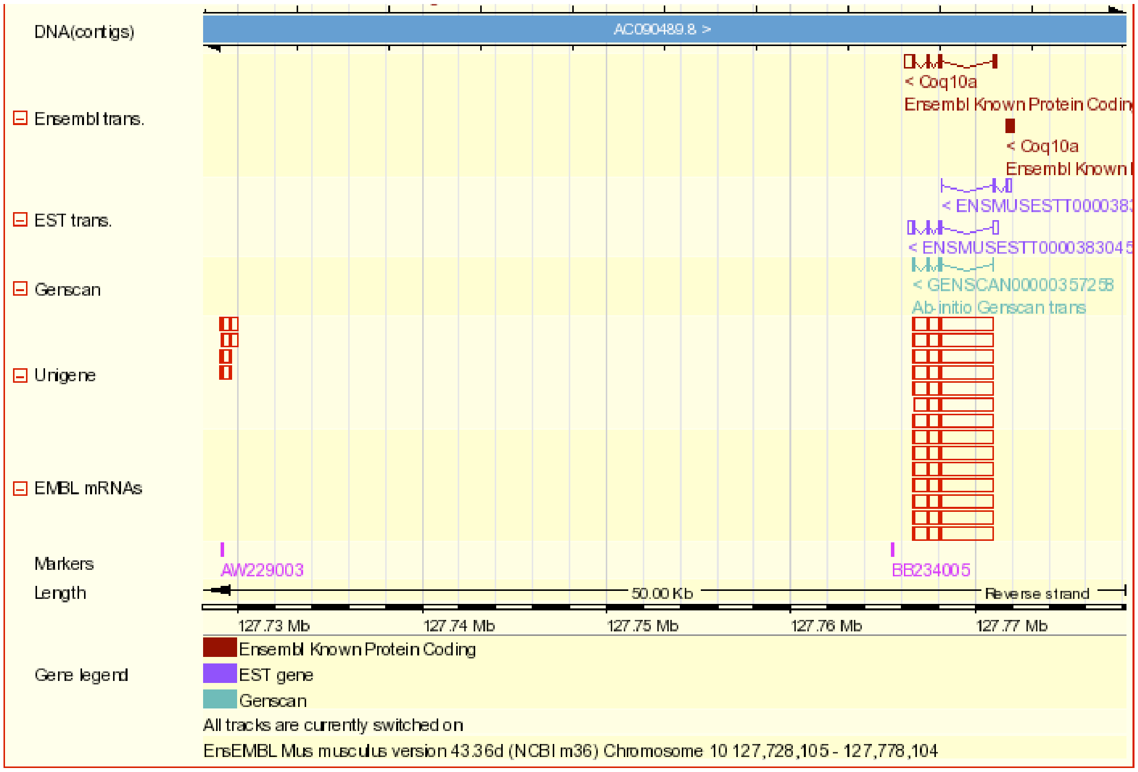

(b) Ribosomal protein L24-like homolog ENSP00000269477 *(Human)*

PR (ENSP00000269477)

[P_{RFC} >0.01 ; Ka/Ks (PR-parent) = 0.75; Ka = 0.07; Ks = 0.10; no match to full-length cDNA]

Chromosome 18

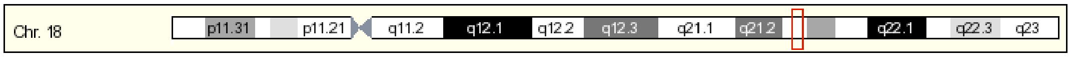

Overview

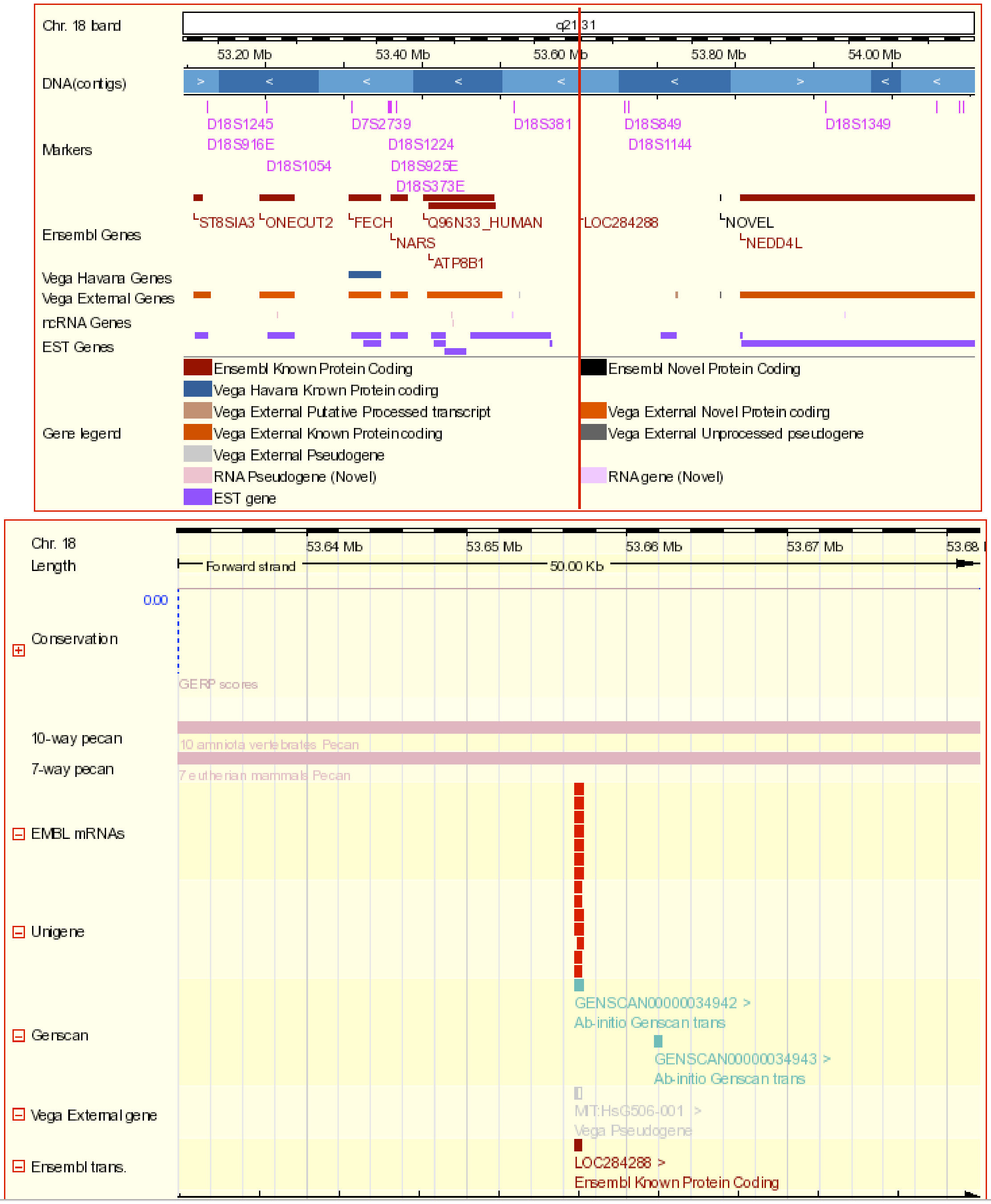

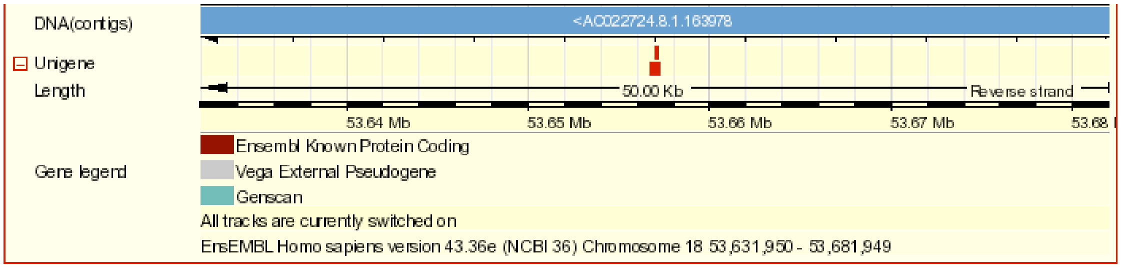

Parent (ENSP00000260443)

Chromosome 15

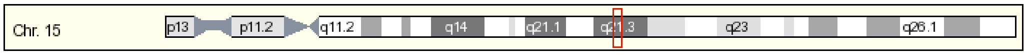

Overview

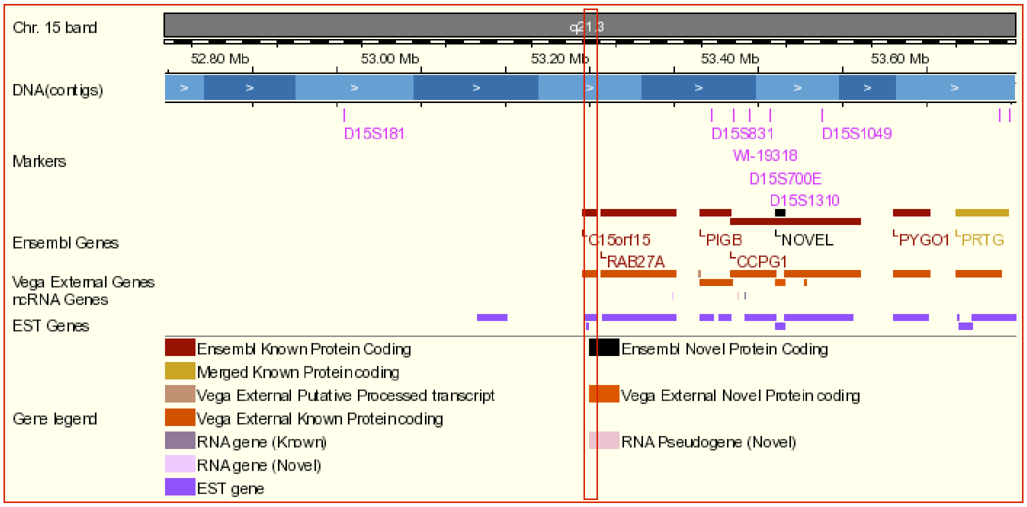

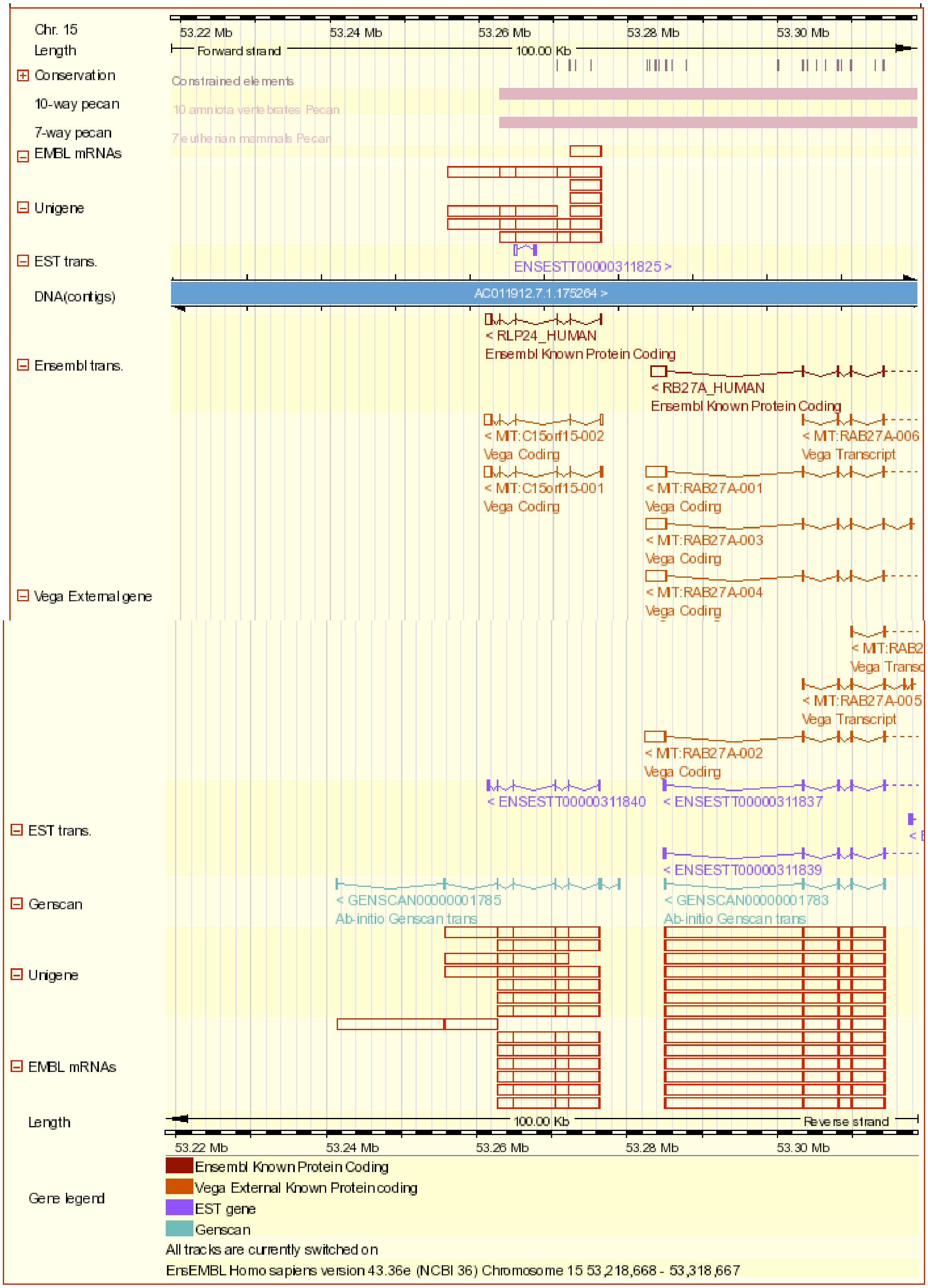

Chr. 15
Length
Conservation
10-way pecan
7-way pecan
EMBL mRNAs
Unigene
EST trans.
DNA(contigs)
Ensembl trans.
Vega External gene
EST trans.
Genscan
Unigene
EMBL mRNAs
Length
Gene legend
53.22 Mb
53.24 Mb
53.26 Mb
53.28 Mb
53.30 Mb
Forward strand
100.00 Kb
Constrained elements
10 amniota vertebrates Pecan
7 eutherian mammals Pecan
ENSESTT00000311825 >
AC011912.7.1.175264 >
< RLP24_HUMAN
Ensembl Known Protein Coding
< RB27A_HUMAN
Ensembl Known Protein Coding
< MIT:C15orf15-002
Vega Coding
< MIT:RAB27A-006
Vega Transcript
< MIT:C15orf15-001
Vega Coding
< MIT:RAB27A-001
Vega Coding
< MIT:RAB27A-003
Vega Coding
< MIT:RAB27A-004
Vega Coding
< MIT:RAB2
Vega Transc
< MIT:RAB27A-005
Vega Transcript
< MIT:RAB27A-002
Vega Coding
< ENSESTT00000311840
< ENSESTT00000311837
< ENSESTT00000311839
< GENSCAN00000001785
Ab-initio Genscan trans
< GENSCAN00000001783
Ab-initio Genscan trans
Reverse strand
Ensembl Known Protein Coding
Vega External Known Protein coding
EST gene
Genscan
All tracks are currently switched on
EnsEMBL Homo sapiens version 43.36e (NCBI 36) Chromosome 15 53,218,668 - 53,318,667

(c) protein similar to chloride intracellular channel 1 (ENSP00000309706) (Human)

PR *(ENSP00000309706)*

[$P_{RFC} \leq 0.01$; Ka/Ks (PR-parent) = 0.44; Ka = 0.07; Ks = 0.16; no match to full-length cDNA]

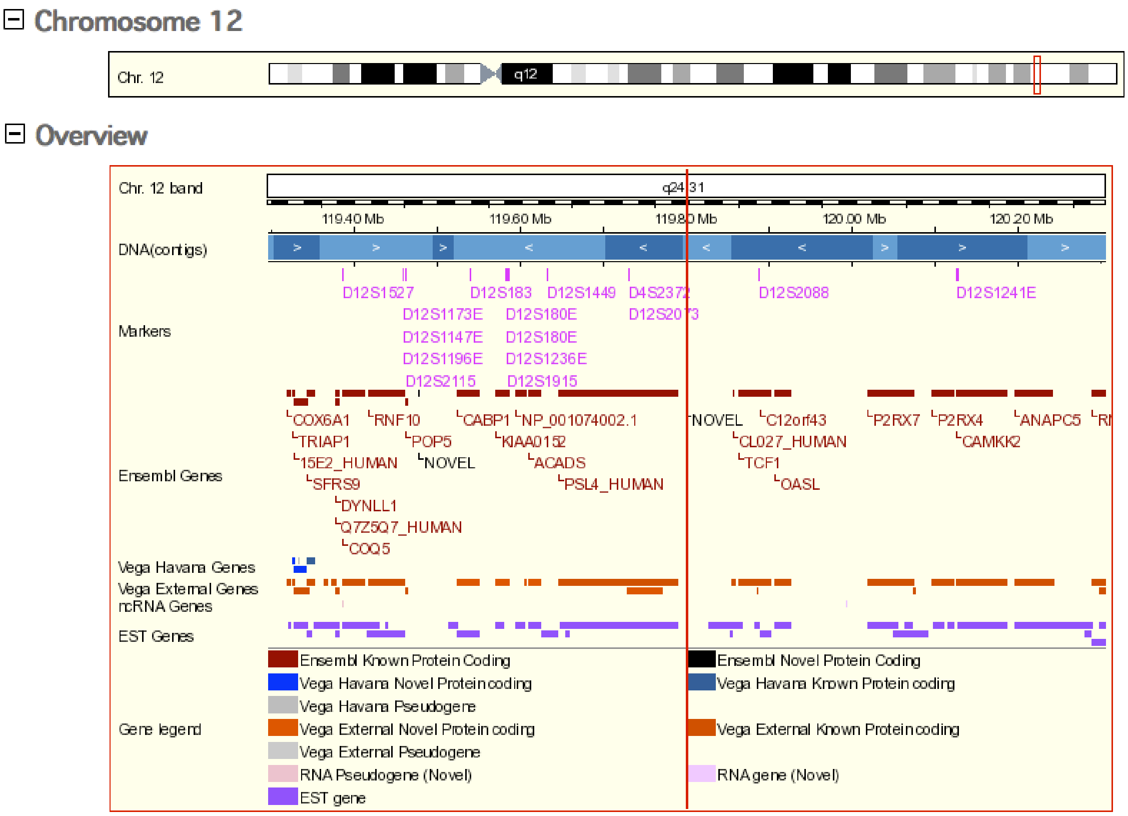

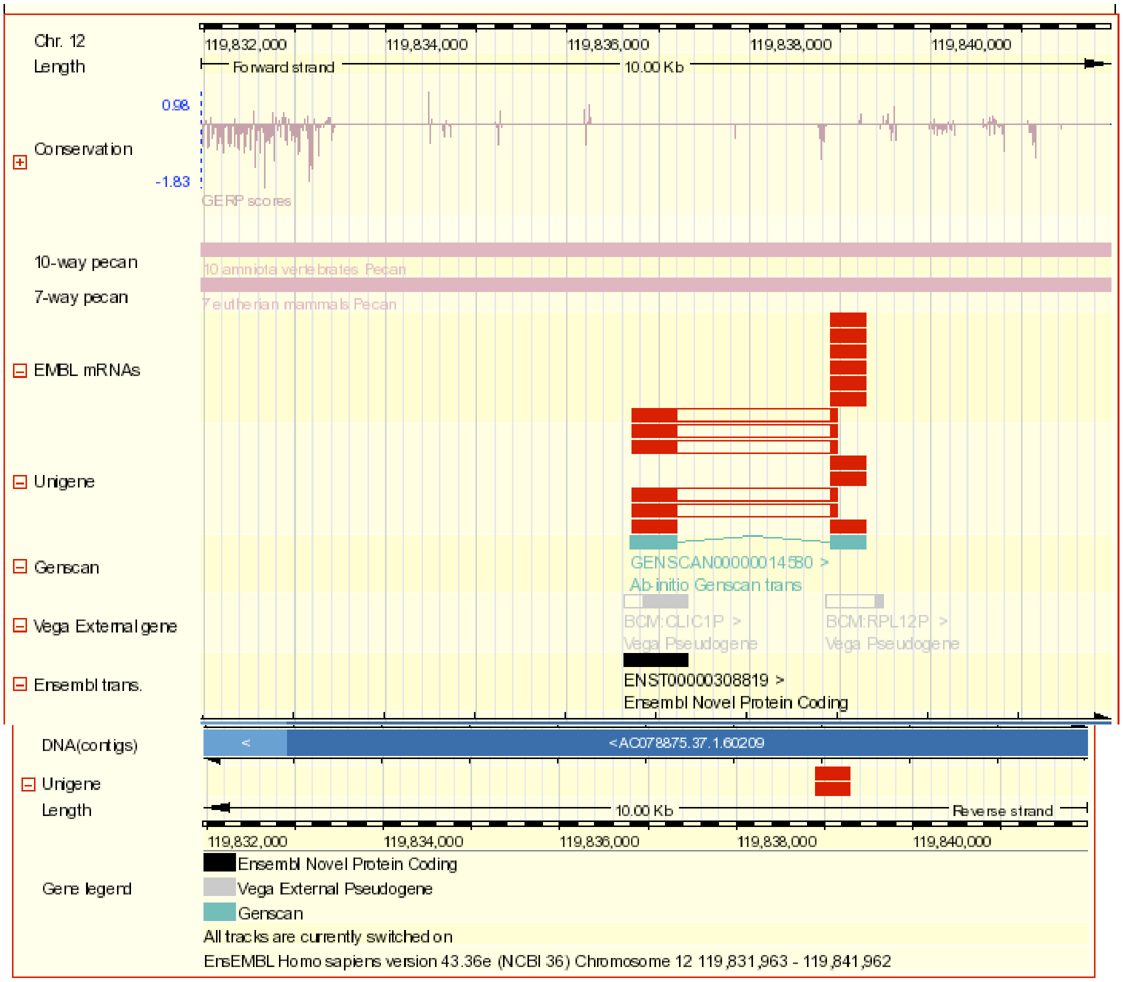

Parent

⊟ Chromosome c6_COX

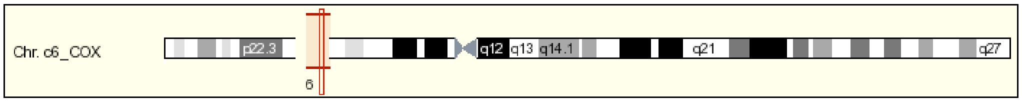

Overview

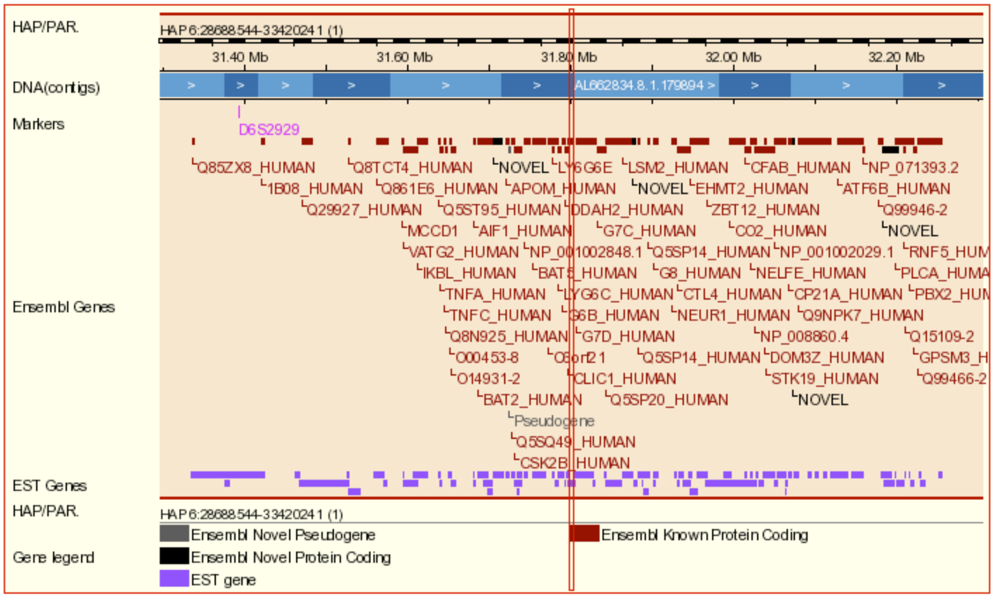

HAP/PAR.
HAP6:28688544-33420241 (1)
31.40 Mb
31.60 Mb
31.80 Mb
32.00 Mb
32.20 Mb
DNA(contigs)
AL662834.8.1.179894 >
Markers
D6S2929
Q8SZX8_HUMAN
Q8TCT4_HUMAN
NOVEL
LY6G6E
LSM2_HUMAN
CFAB_HUMAN
NP_071393.2
1B08_HUMAN
Q861E6_HUMAN
APOM_HUMAN
NOVEL
EHMT2_HUMAN
ATF6B_HUMAN
Q29927_HUMAN
Q5ST95_HUMAN
DDAH2_HUMAN
ZBT12_HUMAN
Q99946-2
MCCD1
AIF1_HUMAN
G7C_HUMAN
CO2_HUMAN
NOVEL
VATG2_HUMAN
NP_001002848.1
Q5SP14_HUMAN
NP_001002029.1
IKBL_HUMAN
BAT5_HUMAN
G8_HUMAN
NELFE_HUMAN
TNFA_HUMAN
LY6G6C_HUMAN
CTL4_HUMAN
CP21A_HUMAN
TNFC_HUMAN
G6B_HUMAN
NEUR1_HUMAN
Q9NPK7_HUMAN
Q8N925_HUMAN
G7D_HUMAN
NP_008860.4
Q15109-2
O00453-8
C6orf21
Q5SP14_HUMAN
DOM3Z_HUMAN
O14931-2
CLIC1_HUMAN
STK19_HUMAN
Q99466-2
BAT2_HUMAN
Q5SP20_HUMAN
NOVEL
Pseudogene
Q5SQ49_HUMAN
CSK2B_HUMAN
Ensembl Genes
EST Genes
HAP/PAR.
HAP6:28688544-33420241 (1)
Ensembl Novel Pseudogene
Ensembl Known Protein Coding
Gene legend
Ensembl Novel Protein Coding
EST gene

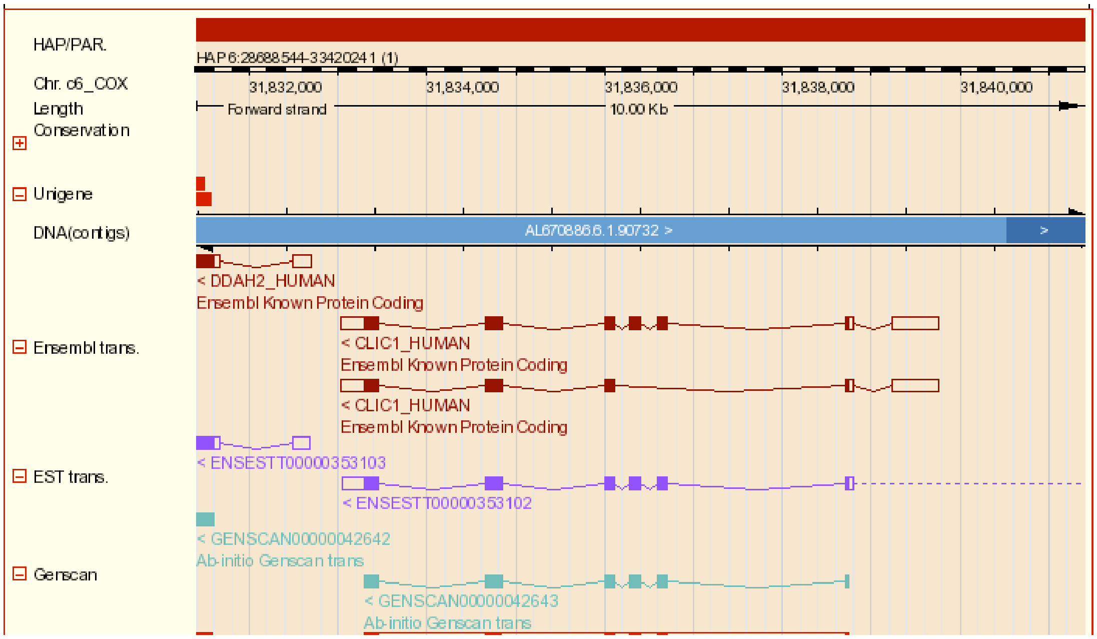

HAP/PAR.
HAP6:28688544-33420241 (1)
Chr. c6_COX
Length
Conservation
31,832,000
31,834,000
31,836,000
31,838,000
31,840,000
Forward strand
10.00 Kb
Unigene
DNA(contigs)
AL670886.6.1.90732 >
< DDAH2_HUMAN
Ensembl Known Protein Coding
Ensembl trans.
< CLIC1_HUMAN
Ensembl Known Protein Coding
< CLIC1_HUMAN
Ensembl Known Protein Coding
< ENSESTT00000353103
EST trans.
< ENSESTT00000353102
< GENSCAN00000042642
Ab-initio Genscan trans
Genscan
< GENSCAN00000042643
Ab-initio Genscan trans

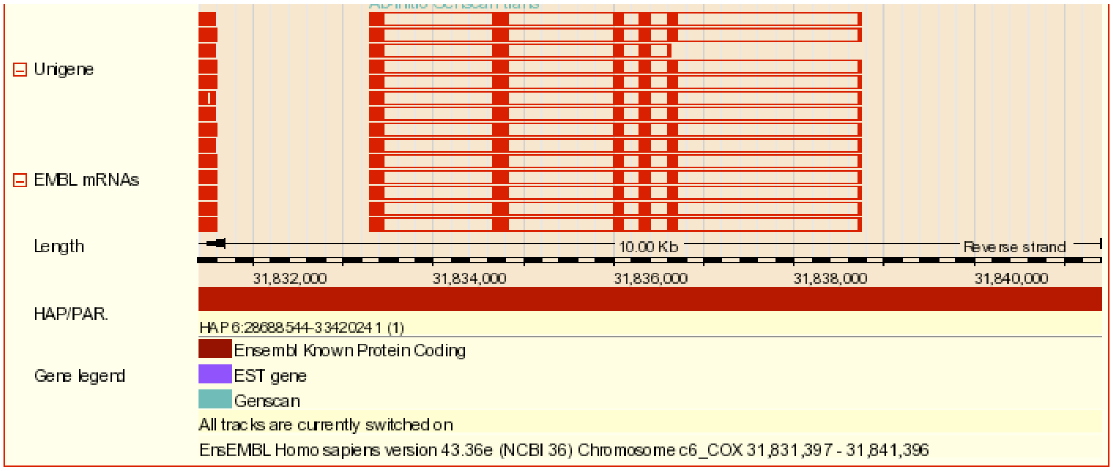

Supplementary Figure 4: Screenshots of three examples of PRs, along with their putative parents. (a) Mouse citrate synthase homolog (ENSMUSP00000052373); (b) Ribosomal protein L24-like homolog ENSP00000269477 (Human); (c) Homolog of chloride intracellular channel 1 (ENSP00000309706) (Human). These images were downloaded from http://www.ensembl.org in April 2007. For each PR, the following are given: the probability of reading-frame conservation (P_{RFC}); the PR-parent Ka/Ks value; the Ka and Ks values and an indication of whether there is a full-length cDNA match.
