# Supplementary material for: Analysis of the role of retrotransposition in gene evolution in vertebrates
Source: BMC Bioinformatics. 2007 Aug 24;8:308. doi: 10.1186/1471-2105-8-308 (PMC2048973; doi:10.1186/1471-2105-8-308)
Supplement: Additional file 5 — Parents with most PRs. The parent genes that spawn most PRs are listed. [file 1471-2105-8-308-S5.doc]

**Supplementary Table 1: Parents with the most PRs ***

(a) Human

| **Number of PRs** | **Parent gene** |
| --- | --- |
| 28 | TRIM41 tripartite-motif-containing 41 (TRI41_HUMAN) |
| 15 | Unnamed protein with EGF-like domains (Q5T669_HUMAN) |
| 6 | Unnamed protein with EGF-like domains (Q9H557_HUMAN) |
| 5 | Ribosomal protein S27 (RS27_HUMAN) |

(b) Mouse

| **Number of PRs** | **Parent gene** |
| --- | --- |
| 6 | Ribosomal protein S29 (RS29_HUMAN) |
| 5 | Translation initiation factor 1 (EIF1_MOUSE) |
| 5 | *SMT3-suppressor-of-mif-two-3* homolog 2 (SUMO2_MOUSE) |

* Those with 5 or more PRs are listed.
